# PRISMA: Protein Interaction Screen on Peptide Matrix Reveals Interaction Footprints and Modifications- Dependent Interactome of Intrinsically Disordered C/EBPβ

**DOI:** 10.1016/j.isci.2019.02.026

**Published:** 2019-03-01

**Authors:** Gunnar Dittmar, Daniel Perez Hernandez, Elisabeth Kowenz-Leutz, Marieluise Kirchner, Günther Kahlert, Radoslaw Wesolowski, Katharina Baum, Maria Knoblich, Maria Hofstätter, Arnaud Muller, Jana Wolf, Ulf Reimer, Achim Leutz

**Affiliations:** 1 Proteome and Genome Research Laboratory, Luxembourg Institute of Health, 1a Rue Thomas Edison, 1445 Strassen, Luxembourg; 2 Max Delbrück Center for Molecular Medicine, Robert-Roessle Strasse 10, 13125 Berlin, Germany; 3 BIH Core Facility Proteomics, Robert-Roessle Strasse 10, 10125 Berlin, Germany; 4 JPT Peptide Technologies GmbH, Volmerstrasse 5, 12489 Berlin, Germany; 5 Humboldt-University of Berlin, Institute of Biology, 10115 Berlin, Germany

**Keywords:** Proteomics, Systems Biology, Transcriptomics

## Abstract

CCAAT enhancer-binding protein beta (C/EBPβ) is a pioneer transcription factor that specifies cell differentiation. C/EBPβ is intrinsically unstructured, a molecular feature common to many proteins involved in signal processing and epigenetics. The structure of C/EBPβ differs depending on alternative translation initiation and multiple post-translational modifications (PTM). Mutation of distinct PTM sites in C/EBPβ alters protein interactions and cell differentiation, suggesting that a C/EBPβ PTM indexing code determines epigenetic outcomes. Herein, we systematically explored the interactome of C/EBPβ using an array technique based on spot-synthesized C/EBPβ-derived linear tiling peptides with and without PTM, combined with mass spectrometric proteomic analysis of protein interactions. We identified interaction footprints of ∼1,300 proteins in nuclear extracts, many with chromatin modifying, chromatin remodeling, and RNA processing functions. The results suggest that C/EBPβ acts as a multi-tasking molecular switchboard, integrating signal-dependent modifications and structural plasticity to orchestrate interactions with numerous protein complexes directing cell fate and function.

## Highlights

• PRISMA yields a high-resolution SLiM- and PTM-based C/EBPβ interactome
• Disclosure of intrinsically disordered protein hub functions and interaction networks
• New connections between C/EBPβ and RNA processing, transcription elongation, MLL, NuRD
• C/EBPβ interaction with TLE3 is regulated by arginine methylation

## Graphical Abstract

**Figure:**
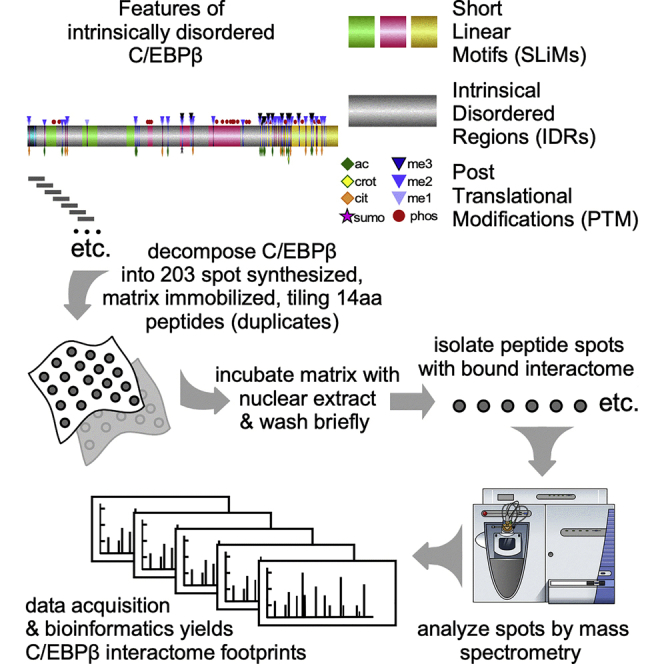

## Introduction

CCAAT enhancer-binding proteins (C/EBPα, β, δ, ɛ) are basic leucine zipper (bZip) transcription factors that regulate chromatin structure and gene expression by dimerization and binding to *cis*-regulatory, palindromic 5′ATTGC·GCAAT3′, or quasi-palindromic DNA sites in gene enhancers and promoters. Prototypic C/EBPβ is widely expressed, highly regulated at the post-transcriptional level, and integrated in many signaling events communicating extracellular cues to epigenetic changes, examples of which include adipogenesis, hematopoiesis, innate immunity, female fertility, skin function, apoptosis, and cellular senescence (Nerlov, 2008, Rodier and Campisi, 2011, Tsukada et al., 2011).

In early hematopoiesis and adipogenesis, C/EBPβ acts as a pioneering factor that orchestrates complex steps in cell fate commitment (Kajimura et al., 2009, Lichtinger et al., 2012, Muller et al., 1995, Ness et al., 1993, Siersbaek et al., 2011). C/EBPβ communicates with numerous other transcription factors, co-factors, histone modifiers, and chromatin remodeling complexes to alter the susceptibility of chromatin to the gene regulatory machinery. C/EBPβ induces lymphoid-myeloid *trans*-differentiation and accelerates acquisition of the induced pluripotent state by the Yamanaka set of reprogramming transcription factors (Di Stefano et al., 2016, Kowenz-Leutz and Leutz, 1999, Stoilova et al., 2013, Xie et al., 2004). The chromatin and gene regulatory functionality of C/EBPβ is linked to distinct regions and post-translational modifications (PTMs) that suspend auto-inhibition, direct the activity of C/EBPβ, and regulate recruitment of chromatin remodelers and writers of histone modifications (Kowenz-Leutz and Leutz, 1999, Kowenz-Leutz et al., 1994, Kowenz-Leutz et al., 2010, Lee et al., 2010b, Mo et al., 2004, Pless et al., 2008, Siersbaek et al., 2011).

C/EBPβ functions are controlled by extracellular signaling cascades involving receptor tyrosine kinases, cytokine receptors, Ras GTPases, mitogen-activated protein kinases, and cyclic AMP and SMAD signaling, and by more complex conditions such as metabolic adaptation, inflammation, senescence, or stress responses (Nerlov, 2008, Rodier and Campisi, 2011, Tsukada et al., 2011). The complexity and diversity of C/EBPβ activities in various cell lineages raises the question of how a single transcription factor can participate in a multitude of regulatory events. Previous research suggested that the combinatorial outcome of post-transcriptional modifications and PTMs in conjunction with intrinsic structural plasticity enables the C/EBPβ protein to adopt a plethora of context- and signal-dependent states that facilitate a variety of interactions (Leutz et al., 2011, Nerlov, 2008, Tsukada et al., 2011).

Post-transcriptional regulation of C/EBPβ generates three isoforms (LAP*, LAP, and LIP) by alternative translation initiation of the single-exon C/EBPβ transcript. As consecutive C/EBPβ start sites are positioned in the same reading frame, the isoforms vary in their gene regulatory N-terminal extensions but retain the same C-terminal dimerization and DNA-binding bZip domain (Wethmar et al., 2010). The diversity of C/EBPβ isoforms is further increased by numerous PTMs of amino acid side chains; in addition to phosphorylation of serine, threonine, and tyrosine residues, lysine acetylation and methylation also occurs, as does methylation of arginine. Enzymes responsible for PTM of C/EBPβ include CARM1/PRMT4, G9A/EHMT2, and CREBBP/KAT3A, all of which also serve as epigenetic histone code writers. The decoration of C/EBPβ by PTM alters its capacity to engage in protein-protein interactions (PPI) and to direct cell fate, suggesting that the signal-dependent C/EBPβ modification index reflects integration of various upstream signaling events to adjust its interactome and to determine its gene regulatory and epigenetic capacity (Leutz et al., 2011). The combination of translational modifications and PTMs may thus encrypt the dynamic interactome, with C/EBPβ being the keystone for a wide range of functional outcomes (Lee et al., 2010b, Sebastian et al., 2005, Sterneck et al., 1997, Stoilova et al., 2013).

The C-terminal third of C/EBPα, β, δ, ɛ contains highly conserved DNA-binding and bZip domains that may dimerize within an extended *trans-*regulatory bZip network including C/EBP, AP-1, and ATF transcription factors. The N-terminal two-thirds region of the C/EBP primary structure is predicted as an intrinsically disordered region (IDR) (Miller, 2006). Phylogenetic analysis, nevertheless, suggests that the C/EBP N terminus also contains several highly conserved short peptide regions (CRs) that are enriched in amino acids with hydrophobic and bulky side chains. These CRs are discontinuous and separated by less conserved and family-specific regions of low complexity characterized by a predominance of small and polar amino acids (Leutz et al., 2011, Tsukada et al., 2011). Experimental studies involving a large number of deletions and CR/IDR shuffling mutants suggested a highly modular, context-dependent functionality of N-terminal C/EBPβ CRs (Kowenz-Leutz et al., 1994, Lee et al., 2010b, Leutz et al., 2011, Williams et al., 1995). Screening for interaction partners using N-terminally derived C/EBPβ peptides further supports the notion that many C/EBPβ interactions may occur in a modular and dynamic fashion that relies on molecular recognition features (MoRFs) and short linear peptide motifs (SLiMs) in combination with adjacent PTMs (Dunker et al., 1998, Leutz et al., 2011, Tompa et al., 2014, van der Lee et al., 2014, Wright and Dyson, 1999).

Based on previous observations of the modular structure and biological functionality of C/EBPβ peptide regions, we developed a novel technique to systematically explore the C/EBPβ interactome. Briefly, C/EBPβ was deconstructed into small linear peptides of 14 amino acids immobilized on a solid-phase matrix. The starting point for each peptide was shifted by four amino acids, creating a tiled peptide array covering the entire amino acid sequence of C/EBPβ. PTM-containing peptides were included according to recorded modifications and their position in the primary C/EBPβ structure. The matrix was incubated with nuclear extracts, and protein enrichment on individual peptide spots was identified and quantified by mass spectrometry. The assay, termed *protein interaction screen on peptide matrices* (PRISMA), revealed hundreds of C/EBPβ- and PTM-specific protein interactions that left footprints on C/EBPβ-derived peptides. Based on comparison with other affinity enrichment approaches, 45 protein complexes were predicted and several novel interactions with proteins and complexes were experimentally confirmed.

## Results

### The C/EBPβ Peptide Matrix

**Figure 1:**
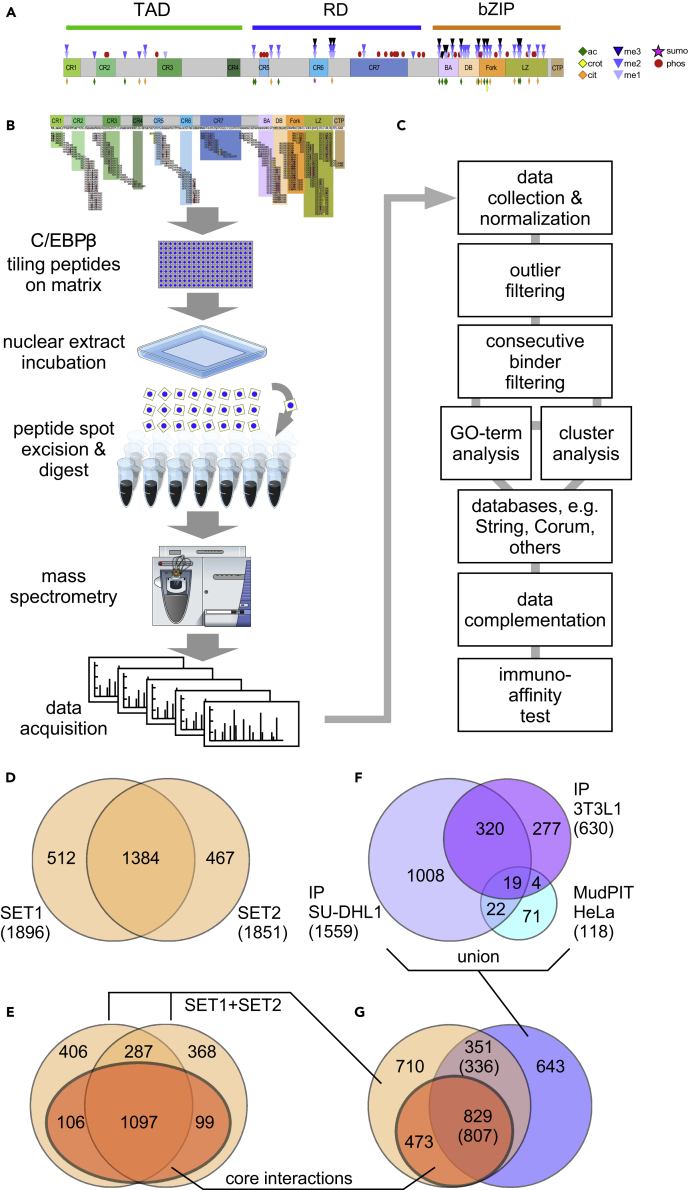
Outline of PRISMA Screen and Comparison of Data (A) Schematic representation of C/EBPβ and the distribution of known post-translational modifications. Conserved regions (CRs) are depicted in color, whereas intrinsic disordered regions are shown in gray. (B) Schematic description of the workflow of protein binding and data acquisition. (C) Workflow of data and proteomic analysis. (D) Overlap between the two replicates (SET1, SET2) of the PRISMA screen. (E) Number of proteins in the two PRISMA datasets that show consecutive peptide binding (core interactions, dark amber). (F) Overlap of three affinity-purification-based datasets of C/EBPβ interactors as described in the literature and combined with data obtained from a proteomic interaction screen in SU-DHL1 cells. Overlaps were determined using immunoprecitation (IP) SU-DHL1 as reference dataset and thus the numbers add up to the size of this set only (see Methods). (G) Overlap of the PRISMA-derived C/EBPβ interactor datasets from (E) (union of SET1 and SET2, light amber) with core interactions (dark amber) from the union of the three datasets from (F) (blue). Overlaps are given using the PRISMA-derived data as reference datasets. The overlap count using the union of the datasets from (F) as a reference dataset is denoted in brackets.

The occurrence of novel PTMs on endogenous C/EBPβ was investigated by mass spectrometry of C/EBPβ immunoprecipitates derived from the human anaplastic lymphoma cell line SU-DHL1 that critically depends for growth and survival on C/EBPβ (Anastasov et al., 2010, Jundt et al., 2005). Over 90 PTMs were identified, which, combined with published data, suggested that more than 130 PTMs may occur on this protein, as summarized in Figure 1A (Table S1). To systematically explore the C/EBPβ interactome and its PTM-specific regulation, we developed the PRISMA technology, which is based on a solid matrix consisting of immobilized peptides spanning the entire primary structure of rat C/EBPβ (297 amino acids), as schematically depicted in Figure 1B and detailed in Table S2. To cover all linear binding regions of the entire CEBPβ protein sequence, tiling peptides 14 amino acids long with an offset of mostly four amino acids were spot synthesized on a cellulose acetate matrix using Fmoc synthesis. As PTMs were previously shown to affect CEBPβ protein interactions and functionality, PTM peptides with S/T/Y-phosphorylation; K-acetylation; K-, R-methylation; and R-citrullination were included in the screen matrix. In total, the solid matrix contained 203 immobilized peptides, covering known and potential post-translational side chain modifications.

### The C/EBPβ PRISMA Screen

To examine the linear CEBPβ interactome, two replicates of the peptide matrix were incubated with nuclear extracts of HeLa cells. HeLa cells express CEBPβ, and commercially available nuclear extracts have been used successfully by many research groups for the purification of biologically active proteins and protein complexes involved in gene regulation (Figure 1B). Individual peptide spots were excised and bound proteins proteolytically digested and analyzed by high-resolution mass spectrometry. In total, 406 analytical mass spectrometric 1-h runs were performed (approximately 17 days of measurement), and spectra were interpreted automatically using the MaxQuant software package.

Enrichment efficiency by PRISMA was examined by comparing the PRISMA intensity distribution with the total proteome of the nuclear extract. Approximately 5,100 proteins were identified and quantified in the nuclear cell extract using the intensity-based absolute quantification method (Schwanhausser et al., 2011). The copy number of proteins in the nuclear cell extract and the number of proteins bound in the PRISMA screen spans six orders of magnitude, suggesting that the identified binders are not biased toward highly abundant proteins (Smits et al., 2013; Figure S1). Furthermore, the distribution of peptide or protein interactions was not attributed to physicochemical parameters of the peptides, as evidenced by comparison of the accumulated interaction intensities with peptide hydrophobicity (gravy index) and isoelectric points, which were determined for all non-modified PRISMA peptides. These results suggest that the PRISMA peptides on the matrix retained their specific protein-binding properties.

### Data Processing of C/EBPβ Peptide-Binding Proteins

An initial inspection of the two replicate datasets revealed signal intensity variation of the interacting proteins. The two datasets were therefore integrated to increase robustness, as outlined in Figure 1C and as explained in the Methods. The main source of variation arose from proteins that were identified only once on different peptide spots. This led to single (low confidence) and double (high confidence) identification categories for each peptide. The signal intensity for each protein was then normalized between 0 and 1 across all 203 matrix peptides. Individual peptides displayed large differences in binding partner profiles, further indicating the specificity of the interactions, because random binding would be expected to result in a more equal distribution of interacting proteins. Further analysis of the data showed that ∼25% of the identified proteins bound to multiple C/EBPβ peptides across the array with low but varying intensity. Although these proteins may promiscuously bind to many distant peptides, some sections of the array showed much higher signals. To minimize noise from background binding, signal intensities for each protein were filtered for binding above 90% of the protein's signal distribution (outlier filtering), removing all signals below this threshold. Another filtering criterion for discriminating the most robust interactors was based on the consecutive binding behavior of tiling peptides of the C/EBPβ primary structure. The rational here is that by shifting the sequence of tiling peptides by four amino acids, some of the SLiMs and fractions thereof are included in more than one peptide. This may generate maximal binding signals for peptides containing optimal SLiM and adjacent supporting amino acids, and attenuated signals from neighboring peptides in which the particular SLiM is shifted or only partially included. We used this predicted binding behavior to stringently filter the dataset further to remove all proteins that failed the consecutive binding criterion (Figures 1D and 1E; see also Methods). In total, 2,363 interacting proteins were identified (Table S3), of which 1,384 proteins were detected in both replicates and 1,302 proteins fulfilled the consecutive binding criterion and were defined as C/EBPβ core interactions (βCI; Figure 1E).

### Validation of the PRISMA-Derived Dataset

To assess the biological significance of data derived by PRISMA, we compared PRISMA data (SET1, SET2, βCI) to previously identified C/EBPβ interactome data, as shown in Figure 1F (Siersbaek et al., 2011, Steinberg et al., 2012). In addition, we performed pull-down experiments using C/EBPβ antibodies and SU-DHL1 cells that express a high amount of CEBPβ and analyzed the CEBPβ interactome by mass spectrometry (Table S4). In total, 1,369 proteins were significantly enriched in SU-DHL1 C/EBPβ samples compared with control samples using a false discovery rate (FDR) cutoff of 5%. The list of SU-DHL1 C/EBPβ interactors was included to extend the existing CEBPβ interaction datasets. The PRISMA core interaction data covered between 38% and 59% of the affinity-purification-based datasets (see Supplemental Information). Affinity purification-based datasets were then combined, and the overlap with PRISMA data was determined on the basis of their UniProt identifier entries, as shown in Figure 1G (see Methods). In total, 47% of data in all three sets were also found in the PRISMA core interactions, and 64% of PRISMA core interactions were also found in at least one of the other datasets. To estimate the FDR of the C/EBPβ interactor data detected by PRISMA, we employed the method proposed previously (D'Haeseleer and Church, 2004), which relies on comparing the intersections of protein interaction datasets to approximate the number of false-positive PPIs. We obtained FDRs of 11.2% and 13.9% for proteins detected in PRISMA replicates 1 and 2, respectively (SET1 and SET2 in Figures 1D and 1E; see Supplemental Information for details). FDRs were reduced to values below 4% when applying the filtering step leading to PRISMA core interactions. These results suggest that PRISMA data depict strong overlap and extensive coverage of the interactome related to native C/EBPβ. We conclude that the PRISMA method successfully extends the interactome data and serves as a resource for locating interaction footprints on C/EBPs.

### High-Resolution C/EBPβ Interactome Footprints

**Figure 2:**
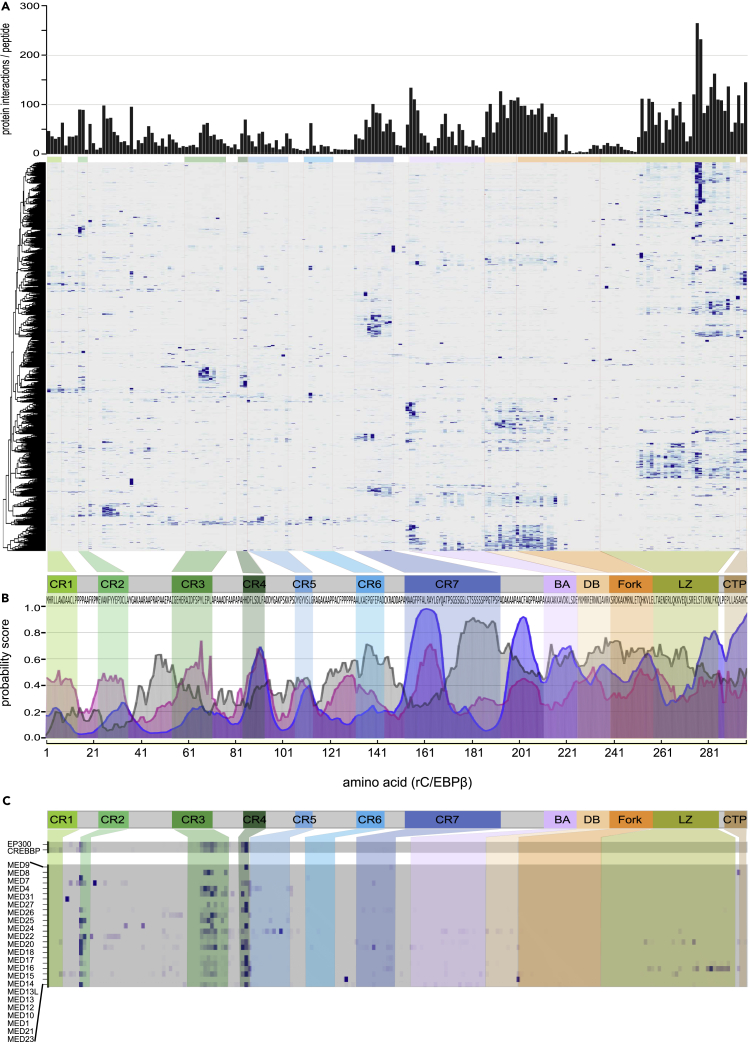
Distribution of Protein Interactions Discovered by PRISMA (A) Heatmap of the normalized intensities of interaction partners. The bar graph (top) shows the distribution of accumulated protein interactions by normalized binding intensities for each peptide. The tiled peptides of C/EBPβ (below) are plotted on the horizontal axis, and proteins are shown on the vertical axis following non-supervised hierarchical clustering. (B) Prediction of C/EBPβ interaction regions using “Anchor” (blue) and “MoRF” (magenta) bioinformatics tools and prediction of intrinsic disorder (gray) in relation to the schematic representation of C/EBPβ and the primary structure (bottom of A). Different conserved regions are colored as shown in Figure 1A. (C) Heatmaps of selected complexes.

The global protein interaction profile obtained from the C/EBPβ peptide matrix is depicted as a non-hierarchically clustered heatmap in Figure 2A. The numerical distribution of proteins identified by individual peptides is shown in the upper part of Figure 2A. Peptides representing the DNA-binding region (DB) and the C-terminal part of the leucine zipper (LZ) exhibit the highest number of protein interactions, yet locally enriched binding hotspots were also found with peptides from the N-terminal part of C/EBPβ.

Clusters of protein-peptide interactions tended to co-localize with regions predicted to undergo disorder-to-order transition during interaction with binding partners and coincide with the profile of conserved C/EBPβ regions (CR1−7), interaction and MoRF predictions (Disfani et al., 2012, Meszaros et al., 2009), as shown in Figure 2B. Predicted IDRs in the transactivating domain (CR1−4), IDR2 (between CR2 and CR3), and regulatory domain between CR7 and the basic or acidic region (IDR7) also displayed large numbers of interaction partners.

Next, PRISMA-derived data were compared with previously mapped C/EBPβ-interacting proteins. As shown in Figure 2C, both co-activator acetyltransferases, CBP and P300 (KAT3A/KAT3B), generated highly similar interaction footprints in the transactivation regions CR4 (strongest binding) and CR3 (additional binding), with some residual binding in CR2. Both C/EBPβ and C/EBPδ have been shown to interact with CBP/p300, and the main interaction region encompasses the CBP Taz2 domain as well as CR3 and CR4 in both C/EBPs. Removal of CR3 or CR4, or mutation of a critical tyrosine residue in CR3 or the DLF motif in CR4 all abrogated interaction with Taz2 and subsequent transcriptional co-activation (Kovacs et al., 2003, Schwartz et al., 2003). Previously published crystallographic data revealed that CR3/CR4 regions in C/EBPɛ may adopt an L-shaped α-helical structure that folds into the p300 Taz2 domain (Bhaumik et al., 2014), in agreement with the CBP/P300 protein footprints revealed by PRISMA (Figure 2C).

The multi-subunit Mediator (MED) complex (Conaway and Conaway, 2013, Jeronimo and Robert, 2017) has also been reported to interact with C/EBPβ (Mo et al., 2004), and several MED components were identified in a previously published C/EBPβ interaction dataset (Siersbaek et al., 2014), as well as in the SU-DHL1 interactome presented here. As shown in Figure 2C, PRISMA revealed 20 components of the multi-subunit MED complex that predominantly interact with CR2-, CR3-, and CR4-derived peptides from C/EBPβ TAD.

CR1 of the LAP* C/EBPβ isoform was reported to specify conjugation by SUMO3 (Eaton and Sealy, 2003) and in accordance, PRISMA showed interaction between SUMO3 and CR1. CR1 also interacts with the Brg1/SMARCA4 ATPase of the SWI/SNF/BAF complex. Moreover, the Brg1-CR1 interaction is sensitive to methylation of arginine 3 (Kowenz-Leutz et al., 2010). Consistent with these results, PRISMA revealed interaction of Brg1 with the unmodified CR1 peptide, but not with the methylated peptide. Moreover, interactions with 10 additional protein components of BAF-SWI/SNF-type protein complexes were detected with various peptides of the C/EBPβ transactivation domain and the bZip domain, indicating multiple interactions between C/EBPβ and chromatin modifying complexes, as previously suggested (Kowenz-Leutz and Leutz, 1999).

### The Landscape of CEBPβ Protein Interactions

PRISMA data (SET1 and SET2) were inspected for enrichment of Gene Ontology (GO) terms, protein families, and protein domains. As shown in Table 1, sorting of C/EBPβ interaction hits based on the quantity of terms resulted in exceedingly low Benjamini-Hochberg corrected FDRs (cutoff at a Benjamini-Hochberg corrected p value of 0.01) (Finn et al., 2016, Finn et al., 2017, Gene Ontology, 2015). Strongly enriched terms included “nucleic acid binding” (745 hits), “gene expression” (611 hits), “protein binding” (534 hits), “regulation of transcription” (301 hits), “cell cycle” (211 hits), “RNA splicing” (172 hits), and several terms involving transcription, chromatin binding, and remodeling. Gene set enrichment analysis showed that approximately 25% of PRISMA-replicate-validated proteins (267) fell into categories that involve GO terms related to RNA processing (FDR 5.63 × 10^−128^). RNA-binding proteins that contain an RNP-1 RNA-binding domain fall into three classes that preferentially interact with the C/EBPβ bZip domain and the adjacent Fork and BA motifs, whereas a third group interacts with the CR2 region. A similar binding pattern was found for DEAD box helicases, a group of enzymes involved in ATP-driven conformational adjustment of ribonucleoprotein assembly.

**Table 1:** C/EBPβ Interactome Enriched Gene Ontology (GO) Terms

Enrichment Score	Pathway/Domain Description	Counts in PRISMA	FDR
**GO: Biological Processes**			

GO:0010467	Gene expression	611	4.19 × 10^−103^
GO:0006355	Regulation of transcription, DNA-templated	301	1.27 × 10^−7^
GO:0006396	RNA processing	267	5.63 × 10^−128^
GO:0007049	Cell cycle	211	3.06 × 10^−31^
GO:0008380	RNA splicing	172	7.14 × 10^−106^
GO:0051276	Chromosome organization	169	1.16 × 10^−34^
GO:0006461	Protein complex assembly	128	4.17 × 10^−12^
GO:0016568	Chromatin modification	89	2.50 × 10^−13^
GO:0051169	Nuclear transport	86	9.46 × 10^−38^
GO:0006281	DNA repair	84	9.48 × 10^−17^

**GO: Molecular Function**			

GO:0003676	Nucleic acid binding	745	3.8 × 10^−199^
GO:0003723	RNA binding	607	0E+00
GO:0005515	Protein binding	534	1.92 × 10^−40^
GO:0003677	DNA binding	269	6.43 × 10^−17^
GO:0005524	ATP binding	216	2.82 × 10^−24^
GO:0003682	Chromatin binding	94	4.43 × 10^−18^
GO:0003712	Transcription cofactor activity	81	2.27 × 10^−9^
GO:0008134	Transcription factor binding	66	5.86 × 10^−9^
GO:0019900	Kinase binding	61	3.30 × 10^−6^
GO:0004693	Cyclin-dependent protein serine/threonine kinase activity	17	6.20 × 10^−10^

**GO: Cellular Component**			

GO:0000785	Chromatin	103	6.04 × 10^−31^
GO:0005681	Spliceosomal complex	85	5.10 × 10^−56^
GO:0005643	Nuclear pore	32	9.77 × 10^−19^
GO:0030880	RNA polymerase complex	30	2.98 × 10^−10^
GO:0070603	SWI/SNF superfamily-type complex	28	9.63 × 10^−14^
GO:0034708	Methyltransferase complex	24	4.86 × 10^−9^
GO:0016592	Mediator complex	20	3.98 × 10^−13^
GO:0000178	Exosome (RNase complex)	12	4.26 × 10^−9^
GO:0016581	NuRD complex	11	1.50 × 10^−9^
GO:0016593	Cdc73/Paf1 complex	6	1.01 × 10^−5^

**Interpro Domains**			

IPR027417	P loop containing nucleoside triphosphate hydrolase	102	9.71 × 10^−30^
IPR000504	RNA recognition motif domain	80	8.77 × 10^−53^
IPR005225	Small GTP-binding protein domain	41	2.11 × 10^−13^
IPR016024	Armadillo-type fold	34	1.69 × 10^−11^
IPR017986	WD40-repeat-containing domain	32	6.78 × 10^−10^
IPR009072	Histone-fold	27	3.89 × 10^−9^
IPR001650	Helicase, C-terminal	23	1.94 × 10^−14^
IPR011989	Armadillo-like helical	21	5.11 × 10^−6^
IPR011545	DEAD/DEAH box helicase domain	20	2.11 × 10^−13^
IPR002041	Ran GTPase	20	5.34 × 10^−12^

**PFAM Domains**			

PF00076	RNA recognition motif. (also called RRM, RBD, or RNP domain)	72	4.32 × 10^−49^
PF00071	Ras family	31	8.17 × 10^−9^
PF00400	WD domain, G-beta repeat	29	1.38 × 10^−10^
PF00271	Helicase-conserved C-terminal domain	23	2.27 × 10^−14^
PF00125	Core histone H2A/H2B/H3/H4	23	1.89 × 10^−9^
PF00270	DEAD/DEAH box helicase	20	2.63 × 10^−13^
PF00013	KH domain	10	1.36 × 10^−6^
PF00244	14-3-3 protein	6	2.32 × 10^−4^
PF01423	LSM domain	9	2.32 × 10^−4^
PF00538	Linker histone H1 and H5 family	5	4.50 × 10^−4^

Protein domain enrichment using InterPro and PFAM databases revealed nucleotide-binding proteins (102 hits), RNA-binding proteins (80 hits), armadillo, WD40, histone fold, and DEAD box domain proteins as major C/EBPβ interaction partners. The number of domain hits obtained using standard algorithms to search protein domain databases may be underrepresented, because manual curation of PRISMA data increased the number of hits for WD40 domain proteins (IPR017986: WD40 repeat, region) from 32 to 40, and the number of DEAD box proteins (IPR011545: DNA/RNA helicase; DEAD/DEAH box type, N-terminal) from 20 to 24.

The C/EBPβ structure is predicted to fold back onto itself, permitting intramolecular signaling, or to stretch out to allow contact with several interaction partners simultaneously (Kowenz-Leutz et al., 1994, Lee et al., 2010a, Lynch et al., 2011). A strong indication of the involvement of multiple contacts with interaction partners through adjacent or distant C/EBPβ regions is shown in Figure S2A, which lists multivalent interactions, including potential interactions with different proteins of the same complex.

Discrete parts of the C/EBPβ primary structure have previously been assigned to different biochemical or cellular functions. Protein binding data from different C/EBPβ regions were extracted and GO term analysis performed for each of the regions to map structure-function relationships, as shown in Figure S2B. Some functional attributions displayed partition over several regions of C/EBPβ (regulation of gene expression, RNA splicing), whereas others are more localized to distinct regions. For example, basic regions and DB show strong enrichment for “regulation of mitotic cell cycle processes” or “nuclear export,” whereas “nuclear import” is associated with CR5, CR7, IR7, and LZ. The C-terminal region of C/EBPβ is involved in the majority of GO terms, whereas transcription factor co-activator functions are mainly associated with N-terminal activation of CR2, CR3, CR4, and IR7.

### C/EBPβ-Interacting Protein Complexes

Next, PRISMA data were analyzed for potential enrichment of large protein complexes interacting with CEBPβ. In addition to anticipated C/EBPβ-interacting complexes such as the basic RNA polymerase II (RNAPII) transcription machinery or MED, previously unknown interrelations became apparent. These included potential complexes involved in the RNA transcript processing machinery responsible for transcript capping, splicing, termination, and polyadenylation. Interactions included 3'- end processing, cleavage, and polyadenylating factor (CPSF1, 3, 7, 30, and 100), and several components of the Integrator complex involved in small nuclear RNA (snRNA) expression and the RNA exosome (EXOSC2, 4, 6, 7, 8, 9, and 10). Furthermore, many components of the nuclear pore and associated adapter complexes were identified. In addition, components of the transcript export THO-TREX complex, including THO1, 2, 3, 5, 6, 7, Aly, DDX39, and the THO-TREX-associated mRNA export factors Nxf1-Nxt1 were identified, along with a large number of heterogeneous nuclear ribonucleoproteins that may couple transcript splicing, maturation, and the formation of messenger ribonucleoprotein particles. Taken together, these data imply the existence of a previously unknown connection between CEBPβ and several steps involved in transcript generation, processing, maturation, and transport (Muller-McNicoll and Neugebauer, 2013, Wickramasinghe and Laskey, 2015).

**Figure 3:**
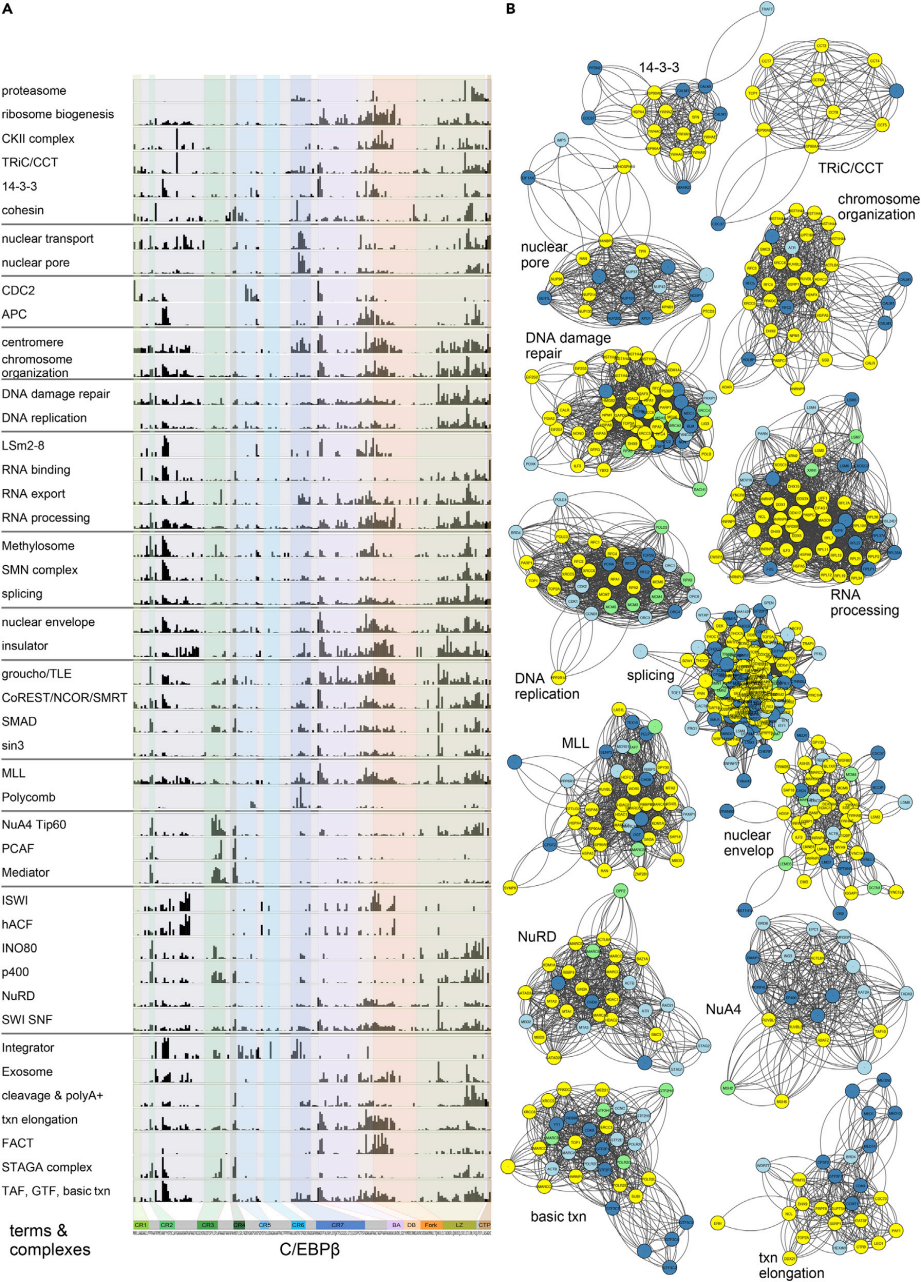
PRISMA-Based Prediction of Protein Complexes Interacting with C/EBPβ (A) Bar graphs of accumulated normalized intensities for 45 selected complexes. Based on CORUM database protein complex annotations, the corresponding normalized intensities of proteins identified by PRISMA were extracted and plotted. (B) Network representation of 14 potential complexes identified. Nodes are color coded according to their detection in PRISMA and SU-DHL1 IP experiments (yellow, detected in IP and PRISMA; green, detected in IP; dark blue, detected in the PRISMA core set; light blue, detected in PRISMA SET1 and SET2).

Proteins co-occurring in both the PRISMA and SU-DHL1 proteomic data were then systematically explored to identify soluble protein complexes listed in the CORUM database (March 6, 2017) using the g:Profiler bioinformatics toolkit (Reimand et al., 2016, Ruepp et al., 2010). A list of 1,432 human protein complexes built from 2,678 proteins (single UniProt identifier; ID) was derived after removal of redundant and non-human complex entries. Proteins listed in PRISMA replicates (SET1 + SET2) and protein interactions derived from SU-DHL1 IP-mass spectrometry data were matched by UniProt and gene name ID using Perseus version 1.5.2.4 (Tyanova et al., 2016). Redundant gene names and isoform entries were merged into a single UniProt ID for each protein. Altogether, 816 proteins of the PRISMA-derived dataset and 490 proteins of the IP SU-DHL1 dataset were included in any of the 1,432 CORUM complexes. The 1,432 CORUM complexes were then ranked by a combination of two criteria: (1) the percentage of proteins in complexes obtained by PRISMA and (2) deviation from randomness of the coverage of the complexes obtained by PRISMA and SU-DHL1 (p values with CORUM background, see Supplemental Information for details). Considering only complexes sharing at least one protein with the PRISMA dataset and which have at least three overlaps with PRISMA and SU-DHL1 data resulted in a ranked list of 417 candidate complexes (see Figure S3 and Table S5). Whenever possible, complexes were grouped into categories indicating functional connections, such as DNA repair, nuclear pore, or centromere, in addition to well-characterized multi-subunit complexes, such as MED, SWI/SNF, MLL, nucleosome remodeling deacetylase (NuRD), and others. As shown in Figure 3A, 45 multi-protein complexes and categories were extracted, and normalized protein interaction values at any of the 203 C/EBPβ peptides were summed over all instances of the category in the list and depicted as a bar plot to visualize the distribution of interaction sites of different categories or complexes in relation to the C/EBPβ primary structure. These 45 entities were composed of 30 complexes or categories representing the full upper quartile of the ranked complex list (i.e., up to rank 104, Table S5, with minor exceptions, Figure S3), together with 15 lower-ranking functional counterparts. Figure 3B shows prominent node-link diagrams of a selection of 14 multi-protein complexes among the highest ranking categories that share many proteins identified by both PRISMA and SU-DHL1 immunoprecipitation methods.

### Validation of Region- and PTM-Specific C/EBPβ Interactions

**Figure 4:**
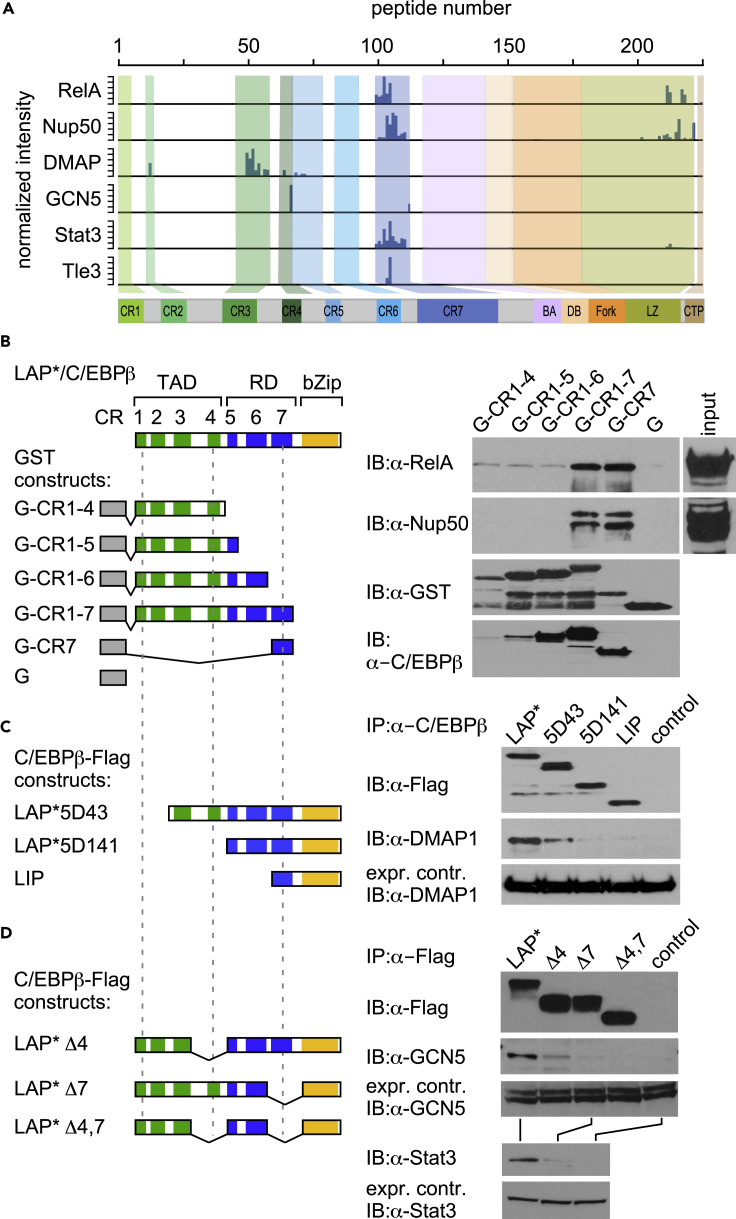
Validation of Site-Specific Interactions with C/EBPβ (A) Accumulated binding intensities of interaction partners (indicated on the left) from the PRISMA screen. Bar graphs indicate interactions according to the peptide position in C/EBPβ, as schematically shown underneath. (B) Top, left: Color-coded scheme of the C/EBPβ protein with dashed lines to aid comparison of constructs shown in (B–D). Top, right: Immunoblots (right) showing interaction of RelA and Nup50/NPAP60L of HEK293T cell lysates with bacterially expressed GST-C/EBPβ constructs. Integrity of GST-C/EBPβ constructs was examined by immunoblotting (as indicated). GST constructs were probed with anti-GST and a polyclonal C/EBPβ antibody that preferentially, but not exclusively, recognizes CR7 as a major epitope. The most intense signals of RelA and Nup50/NPAP60L are associated with the presence of the CR7 region of C/EBPβ. A, antibody; IP, immunoprecipitation; IB, immunoblotting. (C) Co-immunoprecipitation of FLAG-tagged LAP* C/EBPβ or three N-terminal C/EBPβ deletion mutants, followed by immunoblot detection of co-precipitated DMAP1 from HEKT cells. (D) Immunoprecipitation of HEKT cell expressing LAP* and three different internal deletion mutants of C/EBPβ followed by immunoblot detection of GCN5 and Stat3.

Next, conventional protein pull-down, immunoprecipitation, and immunoblotting analysis were used in combination with CEBPβ mutants to validate data from the PRISMA-derived interactome (Figure 4). According to PRISMA, the nuclear factor kappa B subunit RelA, the signal transducer and activator of transcription STAT3, and the nuclear pore complex protein NUP50/NPAP60L predominantly interacted with peptides located in CR7. Bacterially expressed GST-C/EBPβ constructs were probed with HEK293 extracts to examine RelA and NUP50/NPAP60L interactions. As shown in Figures 4B and 4D (lower panels) affinity capture with GST-CEBPβ constructs or co-immunoprecipitation of STAT3 with the C/EBPβ mutant protein lacking CR7 and subsequent immunoblotting confirmed CR7 as the interaction site for all three proteins.

The histone acetyltransferase GCN5 of the SAGA complex and the DNA methyltransferase-associated protein DMAP1 of the NuA4/TIP60 acetyltransferase complex are involved in a wide variety of developmental and genome regulatory activities, including transcription, DNA repair, DNA methylation, and chromatin remodeling (Mohan et al., 2006, Weake and Workman, 2012). DMAP1 and GCN5 engage in major binding to CR3 and CR4, respectively, and minor binding elsewhere in C/EBPβ. Various C-terminally FLAG-tagged C/EBPβ mutant constructs lacking CR1, CR2 (5D43), the entire TAD (5D141), CR4 (LAP*Δ4), CR7 (LAP*Δ7), or both CR4 and CR7 (LAP*Δ4,7) were expressed in HEK293 cells to examine selective and multi-site interaction with resident DMAP1/GCN5 or their respective complexes. As shown in Figures 4C and 4D, pull-down and immunoblotting revealed that removal of the DMAP1 minor binding site in CR2 partially abrogated the interaction and removal of binding to CR3 completely abrogated the interaction. Similarly, removal of the binding site for GCN5 located in CR4 strongly affected GCN5 association and deletion of the binding site in CR7 entirely abolished binding to C/EBPβ.

### The Impact of PTMs on C/EBPβ-Mediated Interactions

**Figure 5:**
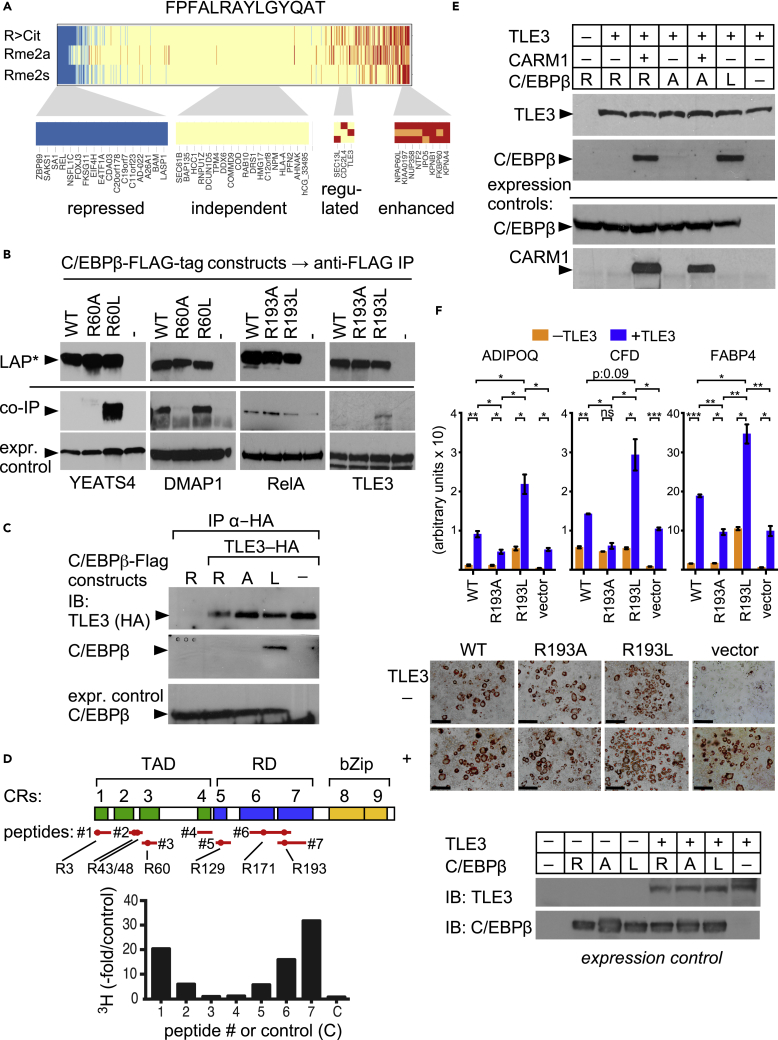
PTM-Specific C/EBPβ Interactions (A) PTM-dependent protein binding to FPFALRAYLGYQAT peptides of C/EBPβ CR7. Normalized binding intensity relative to the unmodified peptide. Four binding categories are considered: proteins with reduced binding by any PTM (repressed, blue, far left), proteins not responding to modifications on C/EBPβ (independent, yellow, middle), proteins with PTM-specific binding (regulated, yellow-red, small), and enhanced binding by any PTM (enhanced, red, far right). (B) Immunoprecipitation (IP) from transfected HEK293T cell lysates: FLAG-tagged C/EBPβ wild-type (WT) or mutants, as indicated, abrogating (C/EBPβ R60A; R193A) or mimicking (C/EBPβ R60L, R193L) methylation. Co-immunoprecipitation (coIP) of endogenous YEATS4 and DMAP1 detected by immunoblotting. YEATS4 and DMAP1 bind to C/EBPβR60L and failed to bind to the R60A C/EBPβ mutant. RelA favors WT or C/EBPβR193A binding over R193L. TLE3 preferentially binds to C/EBPβR193L. Underneath: Expression controls of endogenous YEATS4, DMAP1, RelA, and TLE3. (C) coIP of WT and mutant C/EBPβ, as indicated, with hemagglutinin (HA)-tagged TLE3. C/EBPβR193L, but not WT or R193A, co-IPs with TLE3. (D) *In vitro* methylation of C/EBPβ peptides by CARM1. Scheme of C/EBPβ with approximate positions of peptides and R-residues indicated by red bars and dots; R residue sequence positions underneath. Bar graphs show peptide-specific incorporation of methyl group from donor S-adenosyl-L-[methyl-^3^H] methionine. (E) coIP of C/EBPβ with TLE3 depends on CARM1. Co-expression of WT C/EBPβ (R) or mutants (R193A, R193L), TLE3, and CARM1 are indicated on the top. WT C/EBPβ, but not R193A, co-immunoprecipitates with TLE3 in the presence of CARM1, whereas the C/EBPβR193L mutation supersedes CARM1 requirement. (F) RT-PCR of ADIPOQ, CFD, and FABP4 expression (arbitrary units: target gene to internal 36B4 expression control) induced by C/EBPβ WT, R193A, R193L, or vector control in the absence or presence of TLE3 in NIH 3T3 L1 cells without adipogenic differentiation cocktail (upper panel; n = 3, ANOVA; *p ≤ 0.05, **p ≤ 0.01, ***p ≤ 0.001; ns: not significant). Oil red O staining of stably transfected NIH 3T3 L1 cells with C/EBPβ constructs, vector control and TLE3, as indicated, 3 weeks post-confluency (middle panel, representative images) and protein expression controls (lower panel).

Detection of PTM-dependent protein interactions in linear C/EBPβ peptides was a major objective for the development of the PRISMA screening method. Protein binding to each PRISMA peptide and its PTM-modified versions were therefore examined. The binding signal for each protein was normalized against the signal from the corresponding unmodified peptide to compare enhanced or reduced binding. Interacting proteins were then clustered, as shown in Figure S4. Depending on PTM, as exemplified in Figure 5A, interactions fell into the following four categories: (1) generally repressed binding, (2) PTM-independent binding, (3) PTM-specific binding, and (4) generally enhanced binding. Most of the identified binding partners were indifferent to the type of PTM and were classified as binding proteins that may recognize parts of the peptide sequences. Generally enhanced binding may in part relate to the fact that many PTMs increase hydrophobicity and may thus enhance interactions non-specifically. The most interesting binding partners are expected to be found in the last two categories and represent proteins that are disturbed by any PTM in the peptide or represent interaction partners that were attracted or repelled by a particular PTM. Examples of the latter are the NuA4/TIP60 complex components DMAP1 and YEATS4, both binding to CR3, or RelA and the transducin-like enhancer protein TLE3 binding to CR7, which depends on the methylation status of arginine residues that were subsequently examined.

The transactivation region CR3 (residues 53–68: AIGEHERAIDFSPYLE) contains an arginine residue that is conserved in C/EBPβ, but not in C/EBPα,δ,ɛ, and was previously found to be methylated (Leutz et al., 2011). The PRISMA data suggested that DMAP1 and YEATS4 binding to CR3 depended on methylation of R59. Another interaction hotspot was mapped to the start site of the highly conserved, alternatively initiated LIP C/EBPβ isoform that also represents part of the regulatory region CR7 (residues 158–170: FPFALRAYLGYQAT). The respective arginine residues in both chicken (R60, R193) and rat (R58, R162) were previously found to be methylated. Mutation of the equivalent amino acid residue to alanine or leucine in chicken C/EBPβ strongly altered transcriptional activity, suggesting that the methylation status of the conserved arginine in CR7 may be critical (Leutz et al., 2011). Wild-type (WT) C/EBPβ, methylation-defective R60A and R193A, and methylation-mimicking R60L and R193L constructs were subjected to co-immunoprecipitation to compare alterations in binding, as shown in Figure 5B. As suggested by PRISMA, interaction of all four proteins with C/EBPβ was sensitive to the amino acid side chain configuration at the respective arginine positions, demonstrating both amino acid and PTM specificities, in addition to evolutionary conservation of chicken and rat C/EBPβ interactions. Binding of YEATS4 or DMAP1 to C/EBPβ was enhanced or tolerated by the R60L exchange, but not the R60A exchange, respectively, confirming the side chain specificity and preference for increased side chain hydrophobicity. RelA binding to the R193A mutant was slightly enhanced when compared with WT or reduced by the R193L mutant, respectively, confirming avoidance of the methylated, more hydrophobic PRISMA peptide. By contrast, the TLE3 co-repressor (Agarwal et al., 2015) favored binding to the R193L mutant, suggesting that the increase in hydrophobicity by methylation of the arginine side chain, but not the positive charge, was important for interaction with TLE3. Pull down of TLE3 by immunoprecipitation confirmed preferential binding of C/EBPβR193L, as shown in Figure 5C. Next, we performed methylation assays with the protein arginine methyltransferase PRMT4/CARM1 and C/EBPβ peptides, as shown in Figure 5D. C/EBPβR193 was identified as a methylation target in CR7, in addition to the previously described R3 in CR1 (Kowenz-Leutz et al., 2010). Co-immunoprecipitation of TLE3 together with WT C/EBPβ was dependent on co-expression of PRMT4/CARM1 (Figure 5E). Importantly, and irrespective of co-expressed PRMT4/CARM1, the methylation-defective C/EBPβR193A mutant failed to bind TLE3, confirming C/EBPβ CR7 arginine side chain methylation as a prerequisite for binding to TLE3.

Both C/EBPβ and TLE3 had been described as important regulators in adipogenesis (Villanueva et al., 2013). As shown in Figure 5F, ectopic expression of C/EBPβ in 3-isobutyl-1-methylxanthine (3T3L1) cells induced partial adipogenic differentiation in the absence of the *in vitro* differentiation cocktail (IBMX, insulin, dexamethasone). Fat cell differentiation was evident by up-regulation of the adipogenic genes ADIPOQ, CFD, and FABP4 (Figure 5F, bar graph panel) and enhanced oil red O staining (below) in local adipogenic nests. Adipogenic gene expression of ADIPOQ and FABP4 was enhanced by the C/EBPβR193L mutant, and TLE3 cooperated with C/EBPβ WT and more strongly with C/EBPβR193L, but not with the C/EBPβR193A mutant, to activate ADIPOQ, CFD, and FABP4. We conclude that PRISMA may uncover functional C/EBPβ PTM-regulated protein-protein interactions to adjust the biology of C/EBPβ.

### Novel Connections between C/EBPβ, Transcription Elongation, MLL, and NuRD

Of particular interest was the observation that many proteins forming part of the machinery involved in RNAPII pausing and elongation were included in the PRISMA data. Components of the Trithorax MLL/Set1/COMPAS histone H3 lysine 4 (H3K4) methyltransferase complexes (Shilatifard, 2012) were also represented in the dataset, raising the possibility of a connection between C/EBPβ, enhancer/promoter binding, and regulation of RNAPII processivity. Many components implicated in both processes were found, including the MLL complex components CHD3 and 4 (Mi-2), ASH2, Dpy30, PTIP, and HCF-1; the general transcriptional elongation factor B (TFIIS) and associated elongin A/B/C factors (Aso et al., 1995); the P-TEFb components cyclinK/T1, cdk9, and additional regulatory components including LARP7, HEXIM1, SPT5 (DSIF), and the chromatin adaptor bromodomain factor Brd4 and components of the super elongation complex (SEC), including AF9, PCAF1, and AF4 (AFF1) (Luo et al., 2012). AFF1 is a central SEC scaffold component, and AF9 and ENL are highly similar YEATS domain family members that compete for binding to AFF1. The N-terminal YEATS domain of AF9 and ENL both bind to the PAF complex to connect SEC to RNAPII on chromatin templates. The CDK9/CYCT1 P-TEFb complex is required for rapid transcriptional induction, phosphorylation of the C-terminal domain of RNAPII, and engagement of BRD4, which also interacts with H3K9ac. P-TEFb is also associated with a 7SK snRNA subcomplex that contains the regulatory components LARP7, HEXIM1, and SPT5 (DSIF) and connects to PAFc in the dynamic transcription elongation machinery (He et al., 2011, Luo et al., 2012, Peterlin and Price, 2006). In addition, the histone chaperone FACT complex facilitates passage of the transcription apparatus through chromatin and is thought to restore the chromatin structure and the epigenetic state during transcription, replication, and repair (Hammond et al., 2017, Hondele and Ladurner, 2013, Orphanides et al., 1998).

**Figure 6:**
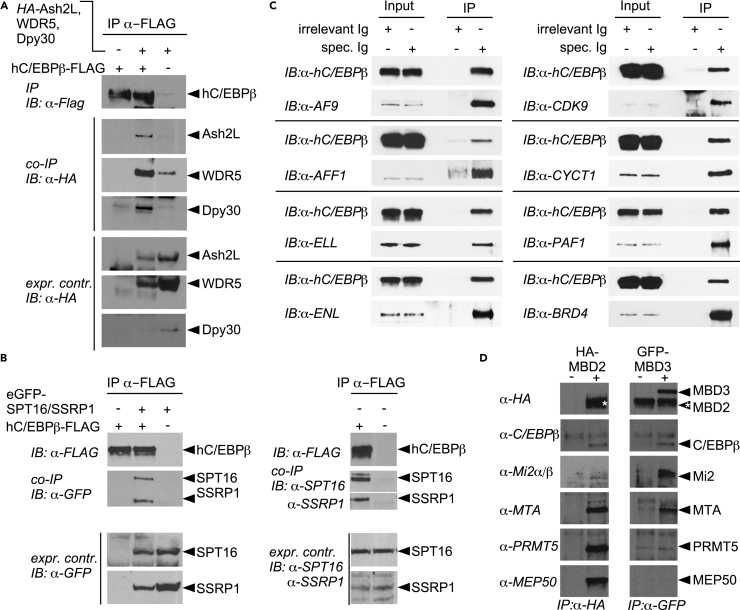
C/EBPβ-Interacting Multi-protein Complexes in HEK293T Cells (A) Co-immunoprecipitation of FLAG-tagged C/EBPβ and the MLL/Ash2/Wdr5 complex. (B) Left panel: FLAG-tagged C/EBPβ was co-expressed with GFP-tagged Spt16 and SSRP1. Right panel: Immunoprecipitation of FLAG-tagged C/EBPβ co-precipitates both endogenous FACT complex subunits. (C) Interaction with the super elongation complex. Immunoprecipitation of human C/EBPβ (C/EBPβ) pulls down the entire super elongation complex. (D) Interaction with the NuRD complex. The integral subunits of the NuRD complex MBD2 (HA-tagged) and MBD3 (GFDP-tagged) were pulled down by immunoprecipitation. Detection by western blot shows that C/EBPβ interacts with both subunits. IB, immunoblotting; IP, immunoprecipitation.

Immunoprecipitation and immunoblotting were performed to validate several of these novel connections. As shown in Figure 6, multiple MLL, FACT, and SEC components were all co-immunoprecipitated with C/EBPβ, confirming the predictive capacity of PRISMA. It is also important to note that AFF1, AF9, and ENL are among the most frequent oncogenic fusion partners with the MLL gene product that transforms early hematopoietic progenitors and causes childhood leukemia by short-circuiting enhancer and promoter activation and transcriptional elongation checkpoint controls (Krivtsov and Armstrong, 2007, Shilatifard, 2012, Slany, 2009, Smith et al., 2011).

The Mi2/NuRD corepressor complex is widely expressed and maintains chromatin in a closed state by ATP-dependent chromatin remodeling and histone tail deacetylation. Some of the NuRD components (CHD3/4(Mi2), HDAC1, RBBP4/7, MTA1,2, GATAD2A/p66α, MBD2, MBD3) identified by PRISMA, SU-DHL1 IP, and other approaches (Steinberg et al., 2012, Siersbaek et al., 2014) are shared with the SIN3A and CoREST repression complexes (Gregoretti et al., 2004), and all were implicated by CORUM analysis (Figures 3 and S3, Table S5). Two major NuRD complexes contain the unique methyl-CpG-binding proteins MBD3 or MBD2, of which the latter may also associate with the WDR77(MEP50)/PRMT5 sub-module (Le Guezennec et al., 2006, Zhang and Yinghua, 2011). Immunoprecipitation of either MBD2 or MBD3 revealed association of C/EBPβ, Mi2, and MTA with both MBD components, whereas only MBD2 co-precipitated with the WDR77(MEP50)/PRMT5 sub-module, suggesting that C/EBPβ may associate with both types of NuRD complexes.

## Discussion

In conclusion, the PRISMA technique reveals a comprehensive and PTM-dependent interactome of the disordered C/EBPβ transcription factor hub and permits the detection of footprints of protein interactions and protein complexes. The results presented in this article suggest that C/EBPβ is involved at the nexus between enhancer and promoter regulation, in many aspects of pre- and post-transcriptional control, including initiation and pausing control, splicing and exosomal RNA degradation, polyadenylation, and RNA maturation, and in nuclear export. Previously, we described an array peptide screening system (APS) for PTM-dependent protein interactions that relied on fluorophore-based detection of peptide interactions with bacterially expressed human proteins immobilized on macroarrays. Although it is difficult to compare APS with PRISMA because of major differences in peptide choice, peptide length, biochemical conditions, local concentration of interaction partners, signal-to-noise ratio, or complexity of the protein expression library and requirement of a renaturation step, 35 common APS/PRISMA interaction partners, including Brg1/SMARCA4, were found with an N-terminal APS peptide and a comparable section covered in PRISMA that contained CR1 (Pless et al., 2011).

The high local concentration of peptides in matrix spots may limit free diffusion and overcome the dissociation problem of low-affinity interactions (Ruthenburg et al., 2007). These features permit the detection of weak interactions and interactions with rare protein partners. The interaction screen developed herein is augmented by inclusion of amino acids with modified side chains to enable detection of PTM-dependent interactions on a global scale. Together, the interaction footprints and PTM-dependent data may help in the rational design of mutants to further explore the functional C/EBPβ interactome and aid in the development of pharmacological inhibitors of protein interactions. The PRISMA workflow is applicable to other regulatory proteins and particularly to transcriptional regulators with a high degree of intrinsic disorder, such as Myc- or various Hox proteins. PRISMA may help to discern highly complex and dynamic transcription factor functions that arise through IDR- and PTM-regulated interactions.

PRISMA was developed for analysis of the interactome of proteins with a high degree of intrinsic disorder. Such proteins lack a defined 3D structure, and protein interactions and complex formation are based on SLiMs, PTMs, and intrinsic flexibility. IDRs in conjunction with PTMs may facilitate structural transitions in partner protein exchanges (Wright and Dyson, 2015). Comprehension of the dynamics and context-dependent interactions between structurally flexible SLiMs and various partner proteins is an emerging hallmark of signal transmission and key to understand the regulation of chromatin structure and gene expression (Minde et al., 2017, Tompa et al., 2014, van der Lee et al., 2014, Wright and Dyson, 2015). SLiM interactions often occur with high specificity but low affinity to enable multiple contacts of a dynamic nature, such as occurring during phase separation. Deciphering the functionality of SLiM/MoRF-type protein regions and PTMs in homeostasis and disease can be challenging when using full-length proteins due to functional redundancy and compensatory effects. A variant of PRISMA has meanwhile been employed to detect dysregulated interactions with disease-causing mutations occurring in IDRs (Meyer et al., 2018). PRISMA data presented here thus provide a repository of high-molecular-resolution C/EBPβ interactions and serve as a basis to explore gene regulatory and PTM-modulated C/EBPβ functions.

### Limitations of the Study

Protein interactions can be mediated by binding domains or by smaller interaction motifs. One key aspect of the study is the identification of the interactome of IDRs and SLiMs and binding regulation by PTMs. As the PRISMA technique is based on the use of peptides, the detection of interactions is limited to those that are based on structurally variable SLiMs, as binding directly depends on the amino acid sequence properties. The high local concentrations of the peptides on the membrane permit the detection of low-affinity interactors and leave components of their corresponding protein complexes intact under mild washing conditions. For interactions that are based on recognition of three-dimensional structures, however, the method will probably not be applicable.

## Methods

All methods can be found in the accompanying Transparent Methods supplemental file.

## Supplemental Information

Document S1. Transparent Methods, Figures S1–S4, and Scheme S1

Table S1. C/EBPβ PTMs, Related to Figure 1A

Table S2. PRISMA C/EBPβ Peptides, Related to Figure 1B

Table S3. The PRISMA C/EBPβ Interactome, Related to Figure 1D

Table S4. C/EBPβ Interactome in SU-DHL cells, Related to Figures 1F and 1G

Table S5. PRISMA-Deduced C/EBPβ complexes, Related to Figure 3

## References

[bib1] Agarwal M., Kumar P., Mathew S.J. (2015). The Groucho/Transducin-like enhancer of split protein family in animal development. IUBMB Life.

[bib2] Anastasov N., Bonzheim I., Rudelius M., Klier M., Dau T., Angermeier D., Duyster J., Pittaluga S., Fend F., Raffeld M. (2010). C/EBPbeta expression in ALK-positive anaplastic large cell lymphomas is required for cell proliferation and is induced by the STAT3 signaling pathway. Haematologica.

[bib3] Aso T., Lane W.S., Conaway J.W., Conaway R.C. (1995). Elongin (SIII): a multisubunit regulator of elongation by RNA polymerase II. Science.

[bib4] Bhaumik P., Davis J., Tropea J.E., Cherry S., Johnson P.F., Miller M. (2014). Structural insights into interactions of C/EBP transcriptional activators with the Taz2 domain of p300. Acta Crystallogr. Sect. D Biol. Crystallogr..

[bib5] Conaway R.C., Conaway J.W. (2013). The Mediator complex and transcription elongation. Biochim. Biophys. Acta.

[bib6] D'Haeseleer P., Church G.M. (2004). Estimating and improving protein interaction error rates. Proc. IEEE Comput. Syst. Bioinform. Conf..

[bib7] Di Stefano B., Collombet S., Jakobsen J.S., Wierer M., Sardina J.L., Lackner A., Stadhouders R., Segura-Morales C., Francesconi M., Limone F. (2016). C/EBPalpha creates elite cells for iPSC reprogramming by upregulating Klf4 and increasing the levels of Lsd1 and Brd4. Nat. Cell Biol..

[bib8] Disfani F.M., Hsu W.L., Mizianty M.J., Oldfield C.J., Xue B., Dunker A.K., Uversky V.N., Kurgan L. (2012). MoRFpred, a computational tool for sequence-based prediction and characterization of short disorder-to-order transitioning binding regions in proteins. Bioinformatics.

[bib9] Dunker A.K., Garner E., Guilliot S., Romero P., Albrecht K., Hart J., Obradovic Z., Kissinger C., Villafranca J.E. (1998). Protein disorder and the evolution of molecular recognition: theory, predictions and observations. Pac. Symp. Biocomput..

[bib10] Eaton E.M., Sealy L. (2003). Modification of CCAAT/enhancer-binding protein-beta by the small ubiquitin-like modifier (SUMO) family members, SUMO-2 and SUMO-3. J. Biol. Chem..

[bib11] Finn R.D., Attwood T.K., Babbitt P.C., Bateman A., Bork P., Bridge A.J., Chang H.Y., Dosztanyi Z., El-Gebali S., Fraser M. (2017). InterPro in 2017-beyond protein family and domain annotations. Nucleic Acids Res..

[bib12] Finn R.D., Coggill P., Eberhardt R.Y., Eddy S.R., Mistry J., Mitchell A.L., Potter S.C., Punta M., Qureshi M., Sangrador-Vegas A. (2016). The Pfam protein families database: towards a more sustainable future. Nucleic Acids Res..

[bib13] Gene Ontology C. (2015). Gene ontology consortium: going forward. Nucleic Acids Res..

[bib75] Gregoretti I.V., Lee Y.M., Goodson H.V. (2004). Molecular evolution of the histone deacetylase family: functional implications of phylogenetic analysis. J. Mol. Biol..

[bib14] Hammond C.M., Stromme C.B., Huang H., Patel D.J., Groth A. (2017). Histone chaperone networks shaping chromatin function. Nat. Rev. Mol. Cell Biol..

[bib15] He N., Chan C.K., Sobhian B., Chou S., Xue Y., Liu M., Alber T., Benkirane M., Zhou Q. (2011). Human polymerase-associated factor complex (PAFc) connects the super elongation complex (SEC) to RNA polymerase II on chromatin. Proc. Natl. Acad. Sci. U S A.

[bib16] Hondele M., Ladurner A.G. (2013). Catch me if you can: how the histone chaperone FACT capitalizes on nucleosome breathing. Nucleus.

[bib17] Jeronimo C., Robert F. (2017). The mediator complex: at the nexus of RNA polymerase II transcription. Trends Cell Biol..

[bib18] Jundt F., Raetzel N., Muller C., Calkhoven C.F., Kley K., Mathas S., Lietz A., Leutz A., Dorken B. (2005). A rapamycin derivative (everolimus) controls proliferation through down-regulation of truncated CCAAT enhancer binding protein {beta} and NF-{kappa}B activity in Hodgkin and anaplastic large cell lymphomas. Blood.

[bib19] Kajimura S., Seale P., Kubota K., Lunsford E., Frangioni J.V., Gygi S.P., Spiegelman B.M. (2009). Initiation of myoblast to brown fat switch by a PRDM16-C/EBP-beta transcriptional complex. Nature.

[bib20] Kovacs K.A., Steinmann M., Magistretti P.J., Halfon O., Cardinaux J.R. (2003). CCAAT/enhancer-binding protein family members recruit the coactivator CREB-binding protein and trigger its phosphorylation. J. Biol. Chem..

[bib21] Kowenz-Leutz E., Leutz A. (1999). A C/EBP beta isoform recruits the SWI/SNF complex to activate myeloid genes. Mol. Cell.

[bib22] Kowenz-Leutz E., Pless O., Dittmar G., Knoblich M., Leutz A. (2010). Crosstalk between C/EBPbeta phosphorylation, arginine methylation, and SWI/SNF/Mediator implies an indexing transcription factor code. EMBO J..

[bib23] Kowenz-Leutz E., Twamley G., Ansieau S., Leutz A. (1994). Novel mechanism of C/EBP beta (NF-M) transcriptional control: activation through derepression. Genes Dev..

[bib24] Krivtsov A.V., Armstrong S.A. (2007). MLL translocations, histone modifications and leukaemia stem-cell development. Nat. Rev. Cancer.

[bib25] Le Guezennec X., Vermeulen M., Brinkman A.B., Hoeijmakers W.A., Cohen A., Lasonder E., Stunnenberg H.G. (2006). MBD2/NuRD and MBD3/NuRD, two distinct complexes with different biochemical and functional properties. Mol. Cell. Biol..

[bib26] Lee S., Miller M., Shuman J.D., Johnson P.F. (2010). CCAAT/Enhancer-binding protein beta DNA binding is auto-inhibited by multiple elements that also mediate association with p300/CREB-binding protein (CBP). J. Biol. Chem..

[bib27] Lee S., Shuman J.D., Guszczynski T., Sakchaisri K., Sebastian T., Copeland T.D., Miller M., Cohen M.S., Taunton J., Smart R.C. (2010). RSK-mediated phosphorylation in the C/EBP{beta} leucine zipper regulates DNA binding, dimerization, and growth arrest activity. Mol. Cell. Biol..

[bib28] Leutz A., Pless O., Lappe M., Dittmar G., Kowenz-Leutz E. (2011). Crosstalk between phosphorylation and multi-site arginine/lysine methylation in C/EBPs. Transcription.

[bib29] Lichtinger M., Ingram R., Hannah R., Müller D., Clarke D., Assi S.A., Lie-A-Ling M., Noailles L., Vijayabaskar M.S., Wu M. (2012). RUNX1 reshapes the epigenetic landscape at the onset of haematopoiesis. EMBO J..

[bib30] Luo Z., Lin C., Shilatifard A. (2012). The super elongation complex (SEC) family in transcriptional control. Nat. Rev. Mol. Cell. Biol..

[bib31] Lynch V.J., May G., Wagner G.P. (2011). Regulatory evolution through divergence of a phosphoswitch in the transcription factor CEBPB. Nature.

[bib32] Meszaros B., Simon I., Dosztanyi Z. (2009). Prediction of protein binding regions in disordered proteins. PLoS Comput. Biol..

[bib33] Meyer K., Kirchner M., Uyar B., Cheng J.Y., Russo G., Hernandez-Miranda L.R., Szymborska A., Zauber H., Rudolph I.M., Willnow T.E. (2018). Mutations in disordered regions can cause disease by creating dileucine motifs. Cell.

[bib34] Miller M. (2006). Phospho-dependent protein recognition motifs contained in C/EBP family of transcription factors: in silico studies. Cell Cycle.

[bib35] Minde D.P., Dunker A.K., Lilley K.S. (2017). Time, space, and disorder in the expanding proteome universe. Proteomics.

[bib36] Mo X., Kowenz-Leutz E., Xu H., Leutz A. (2004). Ras induces mediator complex exchange on C/EBP beta. Mol. Cell.

[bib37] Mohan A., Oldfield C.J., Radivojac P., Vacic V., Cortese M.S., Dunker A.K., Uversky V.N. (2006). Analysis of molecular recognition features (MoRFs). J. Mol. Biol..

[bib38] Muller C., Kowenz-Leutz E., Grieser-Ade S., Graf T., Leutz A. (1995). NF-M (chicken C/EBP beta) induces eosinophilic differentiation and apoptosis in a hematopoietic progenitor cell line. EMBO J..

[bib39] Muller-McNicoll M., Neugebauer K.M. (2013). How cells get the message: dynamic assembly and function of mRNA-protein complexes. Nat. Rev. Genet..

[bib40] Nerlov C. (2008). C/EBPs: recipients of extracellular signals through proteome modulation. Curr. Opin. Cell Biol..

[bib41] Ness S.A., Kowenz-Leutz E., Casini T., Graf T., Leutz A. (1993). Myb and NF-M: combinatorial activators of myeloid genes in heterologous cell types. Genes Dev..

[bib42] Orphanides G., LeRoy G., Chang C.H., Luse D.S., Reinberg D. (1998). FACT, a factor that facilitates transcript elongation through nucleosomes. Cell.

[bib43] Peterlin B.M., Price D.H. (2006). Controlling the elongation phase of transcription with P-TEFb. Mol. Cell.

[bib44] Pless O., Kowenz-Leutz E., Dittmar G., Leutz A. (2011). A differential proteome screening system for post-translational modification-dependent transcription factor interactions. Nat. Protoc..

[bib45] Pless O., Kowenz-Leutz E., Knoblich M., Lausen J., Beyermann M., Walsh M.J., Leutz A. (2008). G9a-mediated lysine methylation alters the function of CCAAT/enhancer-binding protein-beta. J. Biol. Chem..

[bib46] Reimand J., Arak T., Adler P., Kolberg L., Reisberg S., Peterson H., Vilo J. (2016). g:Profiler-a web server for functional interpretation of gene lists (2016 update). Nucleic Acids Res..

[bib47] Rodier F., Campisi J. (2011). Four faces of cellular senescence. J. Cell Biol..

[bib48] Ruepp A., Waegele B., Lechner M., Brauner B., Dunger-Kaltenbach I., Fobo G., Frishman G., Montrone C., Mewes H.W. (2010). CORUM: the comprehensive resource of mammalian protein complexes–2009. Nucleic Acids Res..

[bib49] Ruthenburg A.J., Allis C.D., Wysocka J. (2007). Methylation of lysine 4 on histone H3: intricacy of writing and reading a single epigenetic mark. Mol. Cell.

[bib50] Schwanhausser B., Busse D., Li N., Dittmar G., Schuchhardt J., Wolf J., Chen W., Selbach M. (2011). Global quantification of mammalian gene expression control. Nature.

[bib51] Schwartz C., Beck K., Mink S., Schmolke M., Budde B., Wenning D., Klempnauer K.H. (2003). Recruitment of p300 by C/EBPbeta triggers phosphorylation of p300 and modulates coactivator activity. EMBO J..

[bib52] Sebastian T., Malik R., Thomas S., Sage J., Johnson P.F. (2005). C/EBPbeta cooperates with RB: E2F to implement Ras(V12)-induced cellular senescence. EMBO J..

[bib53] Shilatifard A. (2012). The COMPASS family of histone H3K4 methylases: mechanisms of regulation in development and disease pathogenesis. Annu. Rev. Biochem..

[bib54] Siersbaek R., Nielsen R., John S., Sung M.H., Baek S., Loft A., Hager G.L., Mandrup S. (2011). Extensive chromatin remodelling and establishment of transcription factor 'hotspots' during early adipogenesis. EMBO J..

[bib55] Siersbaek R., Rabiee A., Nielsen R., Sidoli S., Traynor S., Loft A., La Cour Poulsen L., Rogowska-Wrzesinska A., Jensen O.N., Mandrup S. (2014). Transcription factor cooperativity in early adipogenic hotspots and super-enhancers. Cell Rep..

[bib56] Slany R.K. (2009). The molecular biology of mixed lineage leukemia. Haematologica.

[bib57] Smith E., Lin C., Shilatifard A. (2011). The super elongation complex (SEC) and MLL in development and disease. Genes Dev..

[bib58] Smits A.H., Jansen P.W., Poser I., Hyman A.A., Vermeulen M. (2013). Stoichiometry of chromatin-associated protein complexes revealed by label-free quantitative mass spectrometry-based proteomics. Nucleic Acids Res..

[bib59] Steinberg X.P., Hepp M.I., Fernandez Garcia Y., Suganuma T., Swanson S.K., Washburn M., Workman J.L., Gutierrez J.L. (2012). Human CCAAT/enhancer-binding protein beta interacts with chromatin remodeling complexes of the imitation switch subfamily. Biochemistry.

[bib60] Sterneck E., Tessarollo L., Johnson P.F. (1997). An essential role for C/EBPbeta in female reproduction. Genes Dev..

[bib61] Stoilova B., Kowenz-Leutz E., Scheller M., Leutz A. (2013). Lymphoid to myeloid cell trans-differentiation is determined by C/EBPbeta structure and post-translational modifications. PLoS One.

[bib62] Tompa P., Davey N.E., Gibson T.J., Babu M.M. (2014). A million peptide motifs for the molecular biologist. Mol. Cell.

[bib63] Tsukada J., Yoshida Y., Kominato Y., Auron P.E. (2011). The CCAAT/enhancer (C/EBP) family of basic-leucine zipper (bZIP) transcription factors is a multifaceted highly-regulated system for gene regulation. Cytokine.

[bib64] Tyanova S., Temu T., Sinitcyn P., Carlson A., Hein M.Y., Geiger T., Mann M., Cox J. (2016). The Perseus computational platform for comprehensive analysis of (prote)omics data. Nat. Methods.

[bib65] van der Lee R., Buljan M., Lang B., Weatheritt R.J., Daughdrill G.W., Dunker A.K., Fuxreiter M., Gough J., Gsponer J., Jones D.T. (2014). Classification of intrinsically disordered regions and proteins. Chem. Rev..

[bib66] Villanueva C.J., Vergnes L., Wang J., Drew B.G., Hong C., Tu Y., Hu Y., Peng X., Xu F., Saez E. (2013). Adipose subtype-selective recruitment of TLE3 or Prdm16 by PPARgamma specifies lipid storage versus thermogenic gene programs. Cell Metab..

[bib67] Weake V.M., Workman J.L. (2012). SAGA function in tissue-specific gene expression. Trends Cell Biol..

[bib68] Wethmar K., Smink J.J., Leutz A. (2010). Upstream open reading frames: molecular switches in (patho)physiology. BioEssays.

[bib69] Wickramasinghe V.O., Laskey R.A. (2015). Control of mammalian gene expression by selective mRNA export. Nat. Rev. Mol. Cell Biol..

[bib70] Williams S.C., Baer M., Dillner A.J., Johnson P.F. (1995). CRP2 (C/EBP beta) contains a bipartite regulatory domain that controls transcriptional activation, DNA binding and cell specificity. EMBO J..

[bib71] Wright P.E., Dyson H.J. (1999). Intrinsically unstructured proteins: re-assessing the protein structure-function paradigm. J. Mol. Biol..

[bib72] Wright P.E., Dyson H.J. (2015). Intrinsically disordered proteins in cellular signalling and regulation. Nat. Rev. Mol. Cell Biol..

[bib73] Xie H., Ye M., Feng R., Graf T. (2004). Stepwise reprogramming of B cells into macrophages. Cell.

[bib74] Zhang, Yinghua L. (2011). The expanding Mi-2/NuRD complexes: a schematic glance. Proteomics Insights.

