# Supplementary material for: PRISMA: Protein Interaction Screen on Peptide Matrix Reveals Interaction Footprints and Modifications- Dependent Interactome of Intrinsically Disordered C/EBPβ
Source: iScience. 2019 Mar 1;13:351–70. doi: 10.1016/j.isci.2019.02.026 (PMC6424098; doi:10.1016/j.isci.2019.02.026)
Supplement: Document S1. Transparent Methods, Figures S1–S4, and Scheme S1 [file mmc1.pdf]

ISCI, Volume *13*

Supplemental Information

PRISMA: Protein Interaction Screen on Peptide Matrix Reveals Interaction Footprints and Modifications-Dependent Interactome of Intrinsically Disordered C/EBPβ

Gunnar Dittmar, Daniel Perez Hernandez, Elisabeth Kowenz-Leutz, Marieluise Kirchner, Günther Kahlert, Radoslaw Wesolowski, Katharina Baum, Maria Knoblich, Maria Hofstätter, Arnaud Muller, Jana Wolf, Ulf Reimer, and Achim Leutz

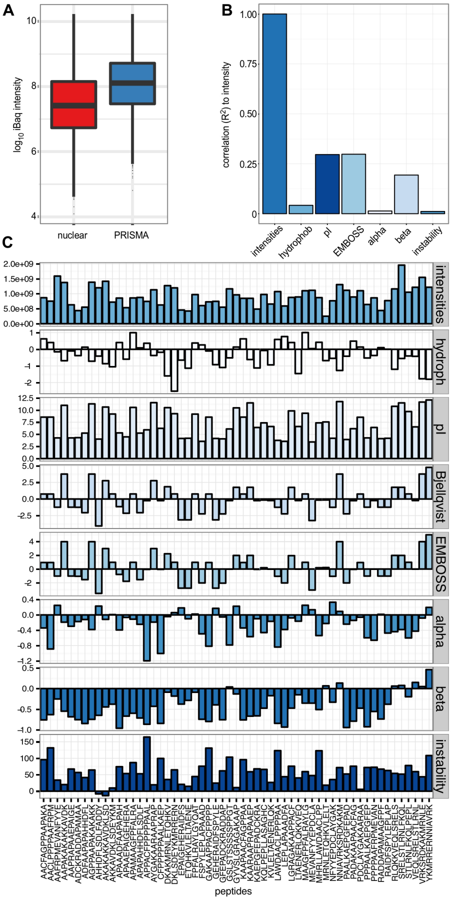

A
log10 iBaq intensity
nuclear
PRISMA
B
correlation (R2) to intensity
intensities
hydrophob
pI
EMBOSS
alpha
beta
instability
C
intensities
hydroph
pI
Bjellqvist
EMBOSS
alpha
beta
instability
peptides

Supplemental Figure 1: Comparison of enrichment by PRISMA and physico-chemical properties of PRISMA peptides. **Related to Figures 1 and 2. A**. Distribution of the copy number. Estimations based on the iBaq quantification for the PRISMA screen and the nuclear extracts. Both, nuclear extracts and PRISMA binders are in the same copy number range. **B**. Correlation of the accumulated intensity of the PRISMA binders to different peptide properties. **C**. Binding of proteins in the PRISMA screen in comparison to various calculated peptide properties. The top panel shows the accumulated binding intensity of the PRISMA binders, while the other panels show the different calculated peptide properties for the peptides listed below.

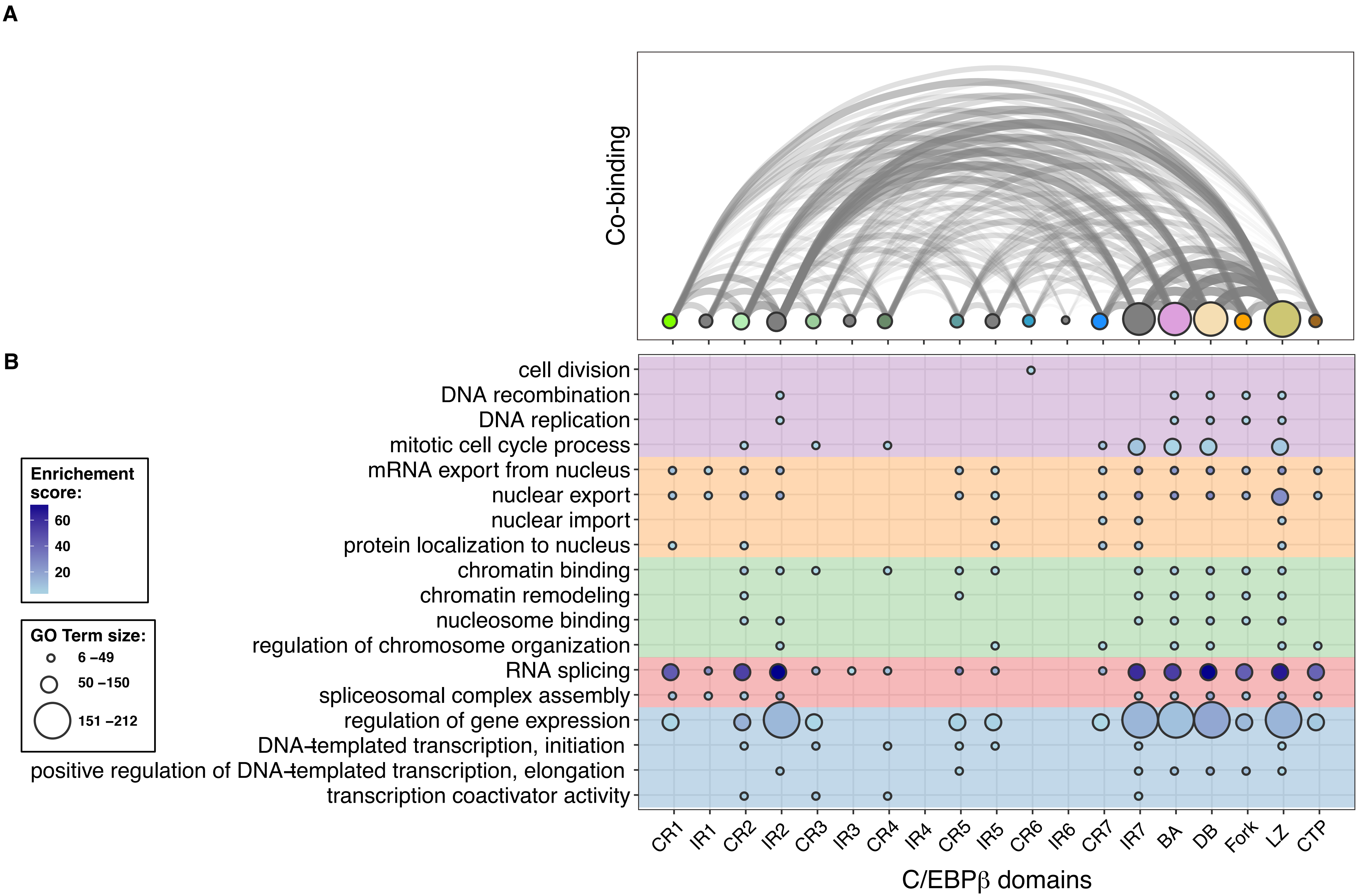
A
Co-binding
B
Enrichement score:
60
40
20
GO Term size:
6 –49
50 –150
151 –212
cell division
DNA recombination
DNA replication
mitotic cell cycle process
mRNA export from nucleus
nuclear export
nuclear import
protein localization to nucleus
chromatin binding
chromatin remodeling
nucleosome binding
regulation of chromosome organization
RNA splicing
spliceosomal complex assembly
regulation of gene expression
DNA-templated transcription, initiation
positive regulation of DNA-templated transcription, elongation
transcription coactivator activity
CR1
IR1
CR2
IR2
CR3
IR3
CR4
IR4
CR5
IR5
CR6
IR6
CR7
IR7
BA
DB
Fork
LZ
CTP
C/EBPβ domains

Supplemental Figure 2: Internal C/EBPβ connections and GO term distribution. Related to Figure 2. A. Co-binding of interaction partners to different C/EBPβ regions. The number of interaction partners was calculated that bind to two different regions, as indicated on the abscissa. Dot sizes represent the number of binding partners in the C/EBPβ regions, while the width of the arcs represent the number of interaction partners found in the connected regions. **B**. Enrichment analysis of GO-terms for binding partners in the different regions of C/EBPβ. GO-terms were selected for DNA related processes (pink), nuclear import/export (orange), chromatin (green), RNA splicing (red) and transcription (blue).

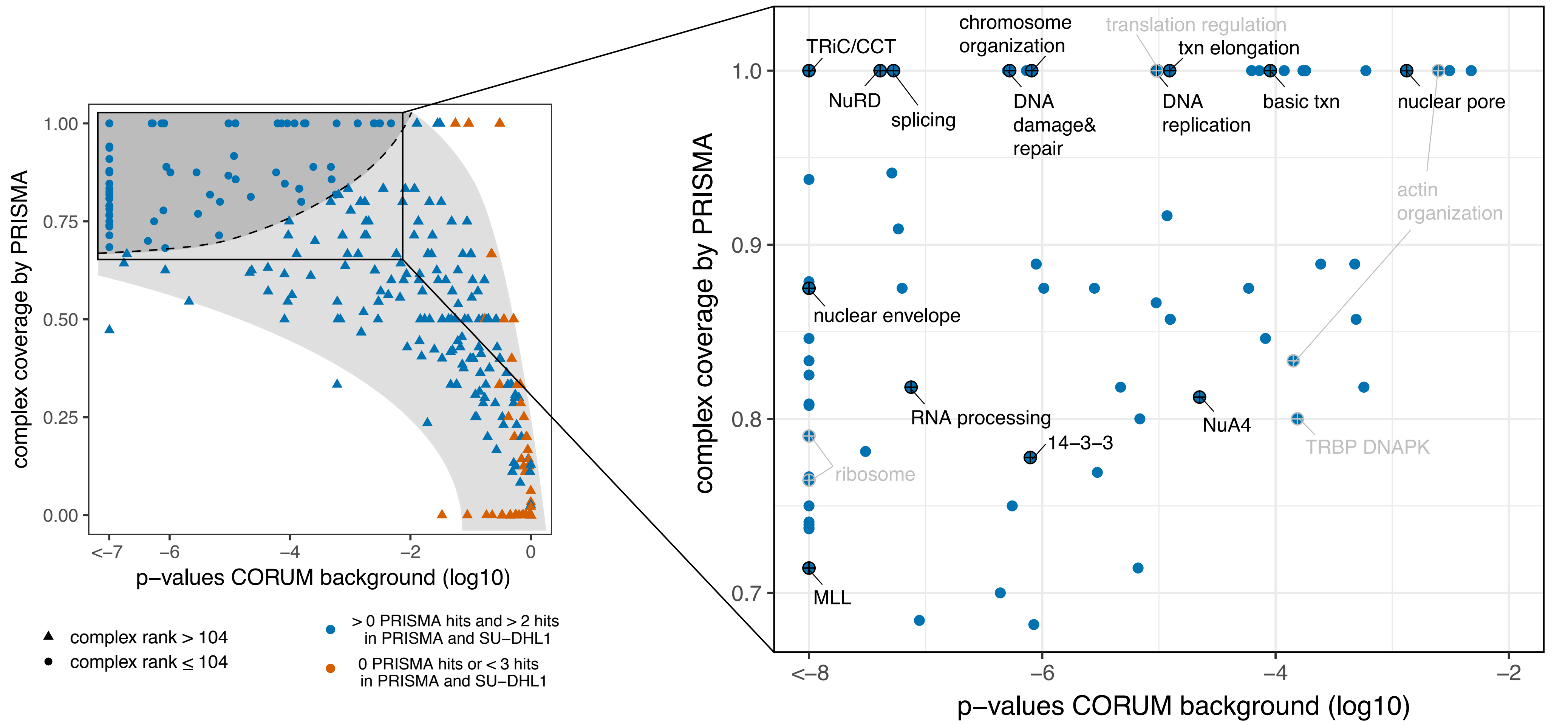

complex coverage by PRISMA
1.00
0.75
0.50
0.25
0.00
<−7
−6
−4
−2
0
p−values CORUM background (log10)
complex rank > 104
complex rank ≤ 104
> 0 PRISMA hits and > 2 hits in PRISMA and SU−DHL1
0 PRISMA hits or < 3 hits in PRISMA and SU−DHL1
TRiC/CCT
chromosome organization
translation regulation
txn elongation
NuRD
splicing
DNA damage& repair
DNA replication
basic txn
nuclear pore
actin organization
nuclear envelope
RNA processing
NuA4
14−3−3
TRBP DNAPK
ribosome
MLL
1.0
0.9
0.8
0.7
<−8
−6
−4
−2

Supplemental Figure 3: Potential C/EBPβ interacting protein complexes. Related to Figure 3. Complex coverage and p-values for each of the 1432 complexes as used for the ranking of CORUM complexes (**Additional Information below**). Each dot corresponds to one complex. Red symbols show complexes which have less than three protein hits in PRISMA and SU-DHL1 data sets. Circles represent complexes ranking within the upper quartile (dark grey) (104 complexes, dashed line between upper quartile and lower ranks), triangles encode lower ranking complexes. The 104 highest ranking complexes are shown in the close-up on the right. Highest ranking complexes for each of the 14 categories from Fig. 4B are indicated in black; other complexes which are not included in Fig. 4A, in grey.

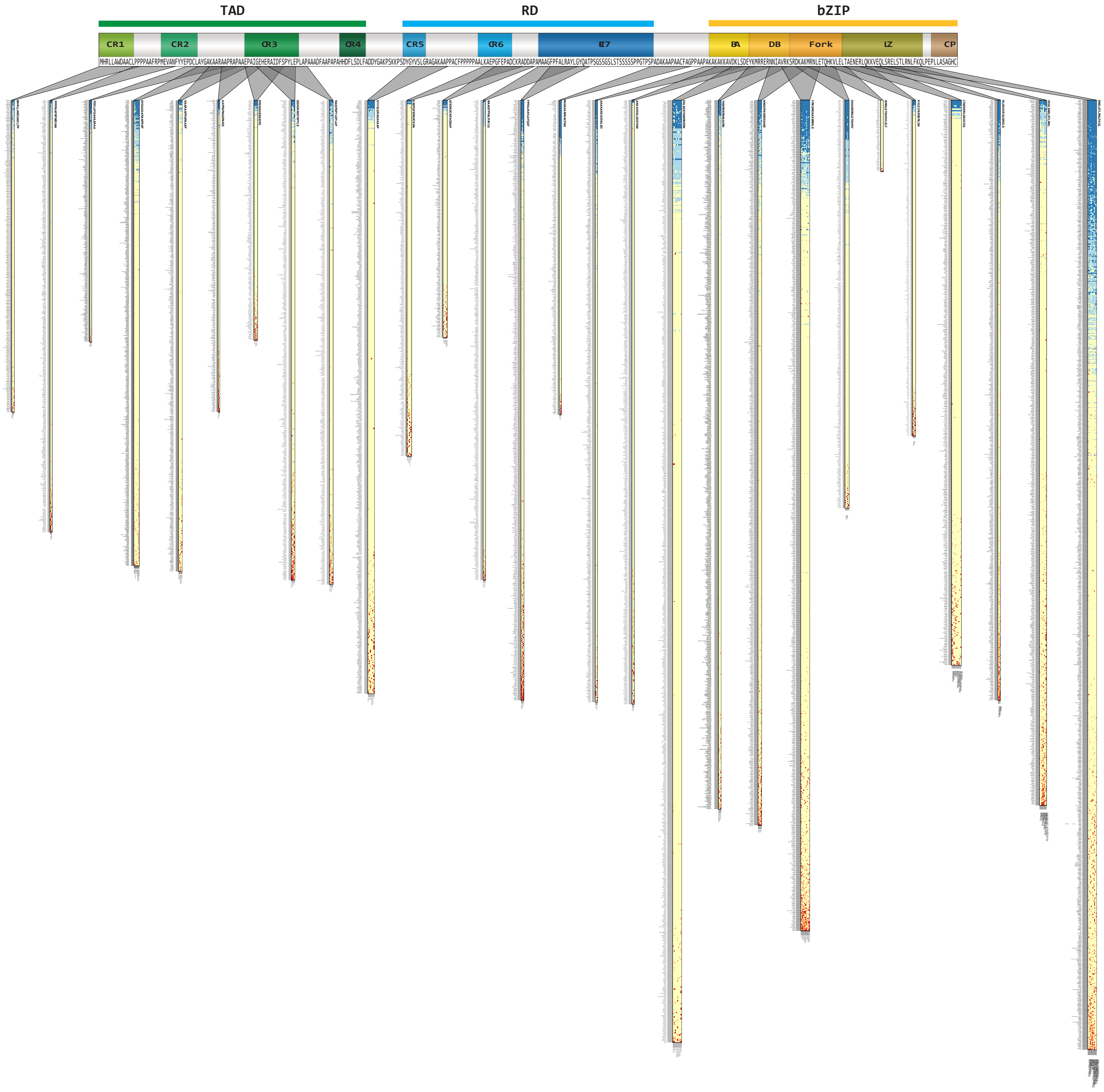
TAD
RD
bZIP
CR1
CR2
CR3
CR4
CR5
CR6
CR7
BA
DB
Fork
LZ
CP

Supplemental Figure 4: PTM-dependent binding across C/EBPβ. Related to Figure 5.

The positions of the PTM-modified peptides are shown on the schematic presentation of the primary C/EBPβ sequence on top. For each peptide, the relative binding to the PTM-modified peptides was calculated in relation to the unmodified peptide. Heatmaps display the relative binding of the proteins on the y-axis in relation to different PTM-carrying peptides, indicated below the heat maps. Red indicates enhanced binding and blue shows reduced binding. Note that the Figure has infinite zoom function for detailed inspection of data.

Transparent Methods

Peptide matrix synthesis

Peptides were synthesized using the automated high-throughput SPOT-synthesis method (Kramer and Schneider-Mergener, 1998). Briefly, Whatman 50 cellulose membranes (Whatman, UK) were functionalized by coupling of Fmoc-protected β-alanine in defined spots. Subsequently, peptides were synthesized stepwise using standard Fmoc-chemistry. After each coupling step before Fmoc-deprotection, peptides that failed coupling of building blocks were acetylated to avoid false sequences.

Interaction screen

The peptide matrix membrane was blocked with yeast tRNA (1 mg/ml; Invitrogen, Karlsruhe, Germany) in binding buffer (20 mM HEPES pH 7.9, Merck, Germany, 0.2 mM EDTA, Merck, Germany, 100 mM KCl Merck, Germany, 20% glycerol, Merck, Germany, 0.5 mM DTT, Merck, Germany) to minimize unspecific protein binding. The membrane was then incubated with HeLa cell nuclear extract (5 mg/ml; Calbiotech S.A, Germany) in binding buffer supplemented with 0.5 mM PMSF (Merck, Germany) for 30 min, then briefly washed with binding buffer. Peptide spots were individually excised and bound proteins converted to peptides using a two-step digest with endopeptidase LysC (Wako, Japan) followed by sequencing-grade trypsin (Promega, Germany) using a robotic setup (Kanashova et al., 2015). Peptide extracts were purified and stored on stage tips (Rappsilber et al., 2007). Two replicates were measured for each peptide spot.

Mass spectrometry measurement for PRISMA

Peptides were cleaned up using a stage-tip micro column (Rappsilber et al., 2007) and resuspended in water with 0.1% formic acid (Merck, Germany). Peptides were separated on a 15cm reverse-phase column (packed in-house, 75 µm inner diameter, 3 µm C_{18}-Reprosil beads; Dr Maisch GmbH, Ammerbruch, Germany) using a gradient to 40% acetonitrile (Merck,

Germany) developed over 1 h 5 min. Separated peptides were ionized on a Proxeon ion source and directly sprayed into the online-coupled VELOS-OrbiTRAP mass spectrometer (Thermo scientific). MS^1 spectra were recorded with a mass resolution of 60,000 in the orbitrap part of the machine. MS^2 spectra were recorded in the VELOS. The ten most intense ions with a charge state greater than 1 were selected (target value = 500; monoisotopic precursor selection enabled) and fragmented in the linear quadrupole trap using CID (collision induced dissociation, 35% normalized collision energy). Dynamic exclusion for selected precursor ions was 60 s. Recorded spectra were analyzed using MaxQuant software package version 1.2.2.5 (Cox and Mann, 2008) and the human IPI database (version 3.3.72), allowing for 2 missed cleavages. Fixed modifications were set to cysteine carbamylation, and variable modifications were set to methionine oxidation, as well as N-terminal protein acetylation. Each replicate was analyzed separately with the label-free option activated for data quantification (Cox et al., 2011).

Analysis of nuclear cell extracts

Nuclear cell extracts were supplemented with the USP2 standard (Merck, Germany) and digested as described above on an automated digestion setup (Kanashova et al., 2015). Peptides were fractionated by RP-HPLC with Proxeon nLC2 and further analyzed by a QExactive mass spectrometer (Thermo Scientific). The mass spectrometer was operated in a data-dependent acquisition mode with dynamic exclusion enabled (30 s). MS^1 (mass range 300-1700 Th) was acquired at a resolution of 70,000 with the ten most abundant multiply charged ($z \geq 2$), ions selected with a 2 Th isolation window for HCD (Higher-energy collisional dissociation) fragmentation. MS^2 scans were acquired at a resolution of 17,500 and injection time of 60 ms.

Full-length C/EBPβ interactome analysis by AP-MS mass spectrometry

Eluates from control immunoglobulins (IgG) and anti-C/EBPβ pull-downs (four replicates each) were ethanol precipitated and protein pellets were solubilised in urea buffer (6 M urea, 2 M thiourea, 20 mM HEPES pH 8), reduced for 30 min at RT in 10 mM DTT, followed by alkylation with 55 mM chloroacetamide (Merck, Germany) for 20 min in the dark at RT. The endopeptidase LysC (Wako, Japan) was added at a protein:enzyme ratio of 50:1 and incubated for 4h at RT. After dilution of the sample with 4× digestion buffer (50 mM ammonium bicarbonate pH 8), sequence-grade modified trypsin (Promega, Darmstadt, Germany) was added (protein:enzyme ratio = 100:1) and digested overnight. Trypsin and LysC activity was quenched by acidification with trifluoroacetic acid (TFA) added to pH ~2. Afterwards, peptides were extracted and desalted using the standard StageTip protocol (Rappsilber et al, 2003). Peptide mixtures were separated by reverse-phase chromatography using an Eksigent NanoLC 400 system (Sciex, Darmstadt, Germany) on in-house-manufactured 20 cm fritless silica microcolumns with an inner diameter of 75 µm. Columns were packed with ReproSil-Pur C18-AQ 3 µm resin (Dr Maisch GmbH). Peptides were separated using an 8–60% acetonitrile gradient (ran over 224 min) at a nanoflow rate of 250 nl/min. Eluting peptides were directly ionised by electrospray ionization and analyzed on a Thermo Orbitrap Fusion instrument (Q-OT-qIT, Thermo). Survey scans of peptide precursors from 300 to 1500 *m*/*z* were performed at 120 K resolution with a 2×10^5 ion count target. Tandem MS was performed by isolation at 1.6 m/z with the quadrupole, HCD fragmentation with a normalized collision energy of 30, and rapid scan MS analysis in the ion trap. The MS^2 ion count target was set to 2×10^3 and the max injection time was 300 ms. Only precursors with a charge state of 2–7 were sampled for MS^2. The dynamic exclusion duration was set to 60 s with a 10 ppm tolerance around the selected precursor and its isotopes. The instrument was run in top speed mode with 3 s cycles, meaning the instrument could continuously perform MS^2 events until the list of non-excluded precursors diminished to zero or 3 s. Data were analyzed by MaxQuant software version 1.5.1.2. The internal Andromeda search engine was used to search MS^2 spectra against a decoy human UniProt database (HUMAN.2014-10) containing forward and reverse sequences. The search

included variable modifications of methionine oxidation and N-terminal acetylation, deamidation (N and Q) and fixed modification of carbamidomethyl cysteine. The minimal peptide length was set to seven amino acids, and a maximum of two missed cleavages were allowed. The FDR was set to 0.01 for peptide and protein identifications. Unique and razor peptides with a minimum ratio count of 1 were considered for quantification. Retention times were recalibrated based on the built-in nonlinear time-rescaling algorithm. MS^2 identifications were transferred between runs with the 'Match between runs' option, in which the maximal retention time window was set to 0.7 min. Statistical analysis was performed using Perseus version 1.5.2.4. C/EBPβ pull-down and control samples were defined as groups and proteins and filtered by intensity value using a 'minimum value of 3 per group' as the threshold. After log2 transformation, missing values were imputed with random numbers from a normal distribution with a mean and standard deviation chosen to best simulate low abundance values below the noise level (width = 0.3; shift = 1.8). Significantly enriched proteins were determined using a volcano plot-based strategy, combining standard two-sample t-test *p*-values with ratio information. Significance corresponding to an FDR of 5% was determined by a permutation-based method (Tusher et al., 2001). Equal sample load was confirmed by calculating the ratio of antibody intensities (mean $\log_2$ ratio = 0.1575).

Identification of C/EBPβ PTM sites by mass spectrometry

Eluates of anti-C/EBPβ pull-downs were ethanol precipitated and protein pellets were processed as described above for the interactome analyses. Peptides were analyzed on a Thermo Orbitrap Fusion instrument (Q-OT-qIT, Thermo). Sequential survey scans of peptide precursors covering different mass ranges (300–600, 550–850, 800–1100, 1050–1700 m/z) were performed at 120 K resolution with a 2×10^5 ion count target on the three most abundant precursor ions. Tandem MS was performed by isolation at 1.6 m/z with the quadrupole, HCD fragmentation with a normalized collision energy of 30, and rapid scan MS analysis in the ion trap. The MS^2 ion count target was set to 1×10^4 and the max injection time was 500 ms. Only precursors with a charge state of 2–7 were sampled for MS^2. The dynamic exclusion duration

was set to 60 s with a 10 ppm tolerance around the selected precursor and its isotopes. Data were analyzed by MaxQuant software version 1.5.1.2 as described above for interactome analysis with some modifications; the search included variable modifications of methionine oxidation and N-terminal acetylation, deamidation (N and Q), phosphorylation (S, T and Y), acetylation (K), methylation (K and R), dimethylation (K and R), trimethylation (K) and citrullination (R). Modification of carbamidomethyl cysteine was set as a fixed modification. The minimal peptide length was set to seven amino acids, and a maximum of four missed cleavages were allowed. The FDR was set to 0.01 for site identifications. To filter for confidently identified peptides, the MaxQuant score was set to a minimum of 40. Identified PTM sites were classified according to their localization probability (Class I >0.75, Class II >0.5, Class 3 >0.25). In order to distinguish citrullination from deamidation, a modification resulting in a similar mass shift of the precursor, both modifications were included during MaxQuant data analyses as variable modifications, and at least one missed cleavage was required for citrullination site identification. When arginine and lysine are acetylated or methylated, trypsin often fails to cleave at that site, resulting in miss-cleaved peptides. Therefore, only the PTM sites, which were identified within the peptide, but not C-terminally localized, were considered as confidently identified.

Combining the two datasets and data filtering

The two datasets of the PRISMA measurement were analyzed in two batches due to restrictions of the MaxQuant software package. The two separate datasets were then integrated by calculating the average intensity value where two values were available, or by taking the single measured value if only one intensity measurement was available to prevent bias against single identifications. Single value intensities were annotated as lower confidence quantifications. Each of the data rows of the combined dataset, corresponding to intensity values of one protein over all 203 peptides, was first filtered according to the outlier criterion $I_n = 0 \; if \; I_n \leq P90$, where I_n is the intensity at peptide n, and P90 is the 90th percentile of the intensity value distribution of the protein, followed by normalization against the intensity of the

highest value in each row using $\frac{I_n}{\max_k(I_k)} = I_n$, where I_n is the intensity at peptide n. This was followed by an additional filtering step where all proteins were removed that did not show a consecutive binding pattern according to finding at least one intensity I_n with $I_n > 0$ and $I_{n+1} > 0$ with I_n as the intensity at peptide n and only considering peptides without any PTM.

Construction of CORUM networks

For each complex identified in the CORUM database, the corresponding UniProt identifier was extracted and translated to an ensemblp identifier using the bioDBnet conversion tool (Mudunuri et al., 2009). For each of the ensemblp identifiers, interactions were extracted from the STRING resource (Szklarczyk et al., 2015). The interaction network was constructed using the igraph software package (Csardi G, 2006) and colored based on the source the of the interaction from the different datasets.

Relative binding to PTM-modified peptides

For each peptide and its PTM derivatives, the relative binding was calculated. For each protein and modified peptide, the intensity value of the unmodified form of the peptide was subtracted. The resulting values were separated into five fractions and clustered according to their Euclidian distance.

Calculation of peptide properties

For calculation of peptide properties, the R peptides package was used, and properties were calculated and plotted using the ggplot2 packages (Wickham, 2009).

Use of the DAVID package

A total of 1375 identifiers were examined using the Generic Gene Ontology Term Finder (GGOTF, Princeton University, Lewis-Sigler Institute for Integrative Genomics) to assess ontology aspects, functions, and components.

Immunoprecipitation of endogenous C/EBPβ for mass spectrometry

Immunoprecipitation of C/EBPβ from 4×10^8 SU-DHL1 cells (anaplastic large cell lymphoma, DSMZ, ACC 356) was performed after washing twice with phosphate-buffered saline (PBS) and resuspension in lysis buffer (20 mM HEPES pH 7.5, 150 mM NaCl, 5 mM $MgCl_2$, 1 mM EDTA pH 8), 1 μM $ZnCl_2$ (Merck, Germany), 0.1% NP40 (Sigma, Germany), 2 mM dithiothreitol (DTT), 2 mM PEFAbloc (Böhringer, Mannheim, Germany) supplemented with protease inhibitor cocktail (Roche, Germany), and 20 U/ml benzonase (Sigma, Germany). After incubation on ice for 10 min, lysates were sonicated twice for 1 min, cell debris was removed by centrifugation at 70,000 g for 30 min, and lysates were filtered through a 0.45 μM filter (Whatman, Maidstone, UK) prior to immunoprecipitation. Samples were immunoprecipitated with a C/EBPβ antibody mix for 30 min at 4°C (Santa Cruz; C-19 and a customized polyclonal antibody raised against the human recombinant C/EBPβ protein) and immunoprecipitates were subsequently collected on Protein-G Dynabeads (Novex, Life Technologies). Beads were washed twice in lysis buffer without benzonase, once in lysis buffer without benzonase and NP40, and eluted by incubation with a mix of 6 M urea and 2 M thiourea (Sigma) for 15 min at 25°C. Immunoprecipitation specificities were controlled by immunoprecipitation of SU-DHL1 lysates with nonspecific rabbit IgG control antibodies (Santa Cruz, sc-2017) and subsequent collection on Protein-G Dynabeads.

Cell culture, transfection, immunoprecipitation and immunoblotting

HEKT-293 cells were grown in Dulbecco's modified Eagle medium (DMEM; Invitrogen, USA) and SU-DHL1 cells were grown in RPMI (Invitrogen, USA) supplemented with 10% FCS and 1% penicillin/streptomycin (Invitrogen, USA). Transfection of plasmids in HEKT-293 cells was performed by calcium-phosphate precipitation or Metafectene (Biontex, Munich, Germany) according to the manufacturer's protocol. For validation of PRISMA-identified C/EBPβ protein interactions, immunoprecipitation of WT or mutant C/EBPβ proteins expressed in HEKT-293 cells was performed as described previously (Kowenz-Leutz et al, 2010). Briefly, cell lysates were prepared in lysis buffer and immunoprecipitation was performed with appropriate antibodies for 2 h at 4°C. Immunoprecipitated proteins were collected on Protein-G Dynabeads (Novex), separated by SDS-PAGE (NuPAGE, Thermo-Fisher, Waltham, USA) and immunoblots were incubated with appropriate antibodies as indicated and visualized by ECL (GE Healthcare, UK). GST-C/EBPβ constructs and cloning of mutant C/EBPβ proteins were as described previously (Kowenz-Leutz et al 1999; 2010 etc). Antibodies were as follows: anti-C/EBPβ (Leutz lab), anti-C/EBPβ (Santa Cruz; C-19), anti-Flag (Sigma), anti-HA.11 (Covance), anti-TLE3 (Santa Cruz; sc-9124), anti-WDR77/Mep50 (Biomol; A301-562A), anti-Nup50 (Santa Cruz; sc-133859), anti-Mi2 (Santa Cruz; sc-11378; Santa Cruz; sc-11378), anti-MBD2 (Santa Cruz; sc-12444), anti-MBD3 (Bethyl; A302-538A), anti-PRMT5 (Millipore; 07-405), anti-MTA1 (Biomol; A200-280A), DMAP1 (Santa Cruz; B-10), anti-RbbP4 (Abcam), Stat3 (Cell signaling; 9132P), anti-RelA (Santa Cruz; sc-109, anti-GCN5 (H-75; Santa Cruz; SC-20698), anti-ELL (Bethyl; A301-644A), anti-cyclin T1 (Bethyl; A303-499A-M), anti-CDK9 (Bethyl; A303-493A-M), anti-AF9 (Novus; NB100-1565), anti-MLLT1 (Novus; NBP1-26653), anti-PAF1 (Novus; (NB600-274), anti-BRD4 (Bethyl; A301-985A50), anti-AF4 (Santa Cruz; sc-99062), anti-GFP (Roche; 11814460001), anti-SSRP1 (Thermo; PA-22186), anti-SPT16 (Thermo; PA1-12697).

In *vitro* methylation assay

Peptides, #1-7 (PSL, Heidelberg, Germany) used for in vitro methylation were based on the mouse C/EBPβ homolog. Peptide sequences are:

#1: MHRLLAWDAASLPPPPAAFRP,

#2: MEVANFYYEPDSLAYGAKAARAAPRAAPAAEPAIG,

#3: AAEPAIGEHERAIDFSPYLEPLAPAADFAAPAP,

#4: APHHDFLSDLFADDYGAKPSKKPADYGYVSLG,

#5: ADYGYVSLGRAGAKAAPPASFP,

#6: PPAALKAEPGFEPADSKRADDAPAMAAGFPFALRAYLGYQATPSG,

#7: MAAGFPFALRAYLGYQATPSGSSGSLSTSSSSSPPGTPSPDADKA.

Methylation assays were carried out with PRMT4/CARM1 (#51047, BPS Bioscience) in the presence of S-adenosyl-L-(methyl)-3H methionine as methyl donor and incorporation of (3H)-methyl was determined by scintillation counting (Kowenz-Leutz et al 2010).

Adipogenesis and RT-PCR analysis

Adipogenic differentiation of was performed with C/EBPβ WT, R193A,L mutant and TLE3 constructs were transfected into 3T3L1 fibroblasts and cells were grown in the absence of the adipogenic differentiation cocktail (IBMX, Insulin, Dexamethasone) (Kowenz-Leutz et al 2010). Total RNA was isolated (#R1055, Zymo) and cDNA was prepared (#K1622, ThermoScientific). Real-time PCR was performed using SYBR Green (#A25741, Applied Biosystems and the Quant Studio 6 Flex Cycler (Applied Biosystems). Primers were used as described (Villanueva et al., 2013). Primer sequences: m36B4 forward AGATGCAGCAGATCCGCAT and reverse GTTCTTGCCCATCAGCACC; ADIPOQ forward CCGGAACCCCTGGCAG and reverse

CTGAACGCTGAGCGATACACA; CFD forward CATGCTCGGCCCTACATGG and reverse CACAGAGTCGTCATCCGTCAC; FABP4 forward CACCGCAGACGACAGGAAG and reverse GCACCTGCACCAGGGC.

Adipogenic cell differentiation was visualized after fixation with 4% paraformaldehyde by oil red O staining.

Homolog mapping and calculation of overlap with known datasets

The two Siersbaek interaction datasets (Siersbaek et al., 2011) are based on the mouse homolog of C/EBPβ and thus all identifiers were mapped to human homologs prior to calculating the overlap between the datasets using the InParanoid Homolog database (http://inparanoid.sbc.su.se/cgi-bin/index.cgi). PRISMA data was organized into protein groups reflecting the non-unique mapping of peptides to the protein sequences. Within PRISMA data, the unique IPI identifiers served for overlap calculation. Otherwise, the overlap of a set of protein groups (reference dataset) with another dataset was calculated by determining all mutual UniProt identifiers in the two datasets and projecting them onto the protein groups of the reference dataset.

Calculations of dataset overlaps

Overlap of proteins in the full PRISMA dataset (replicates 1 and 2), the PRISMA core interaction set, and the three other datasets (MudPIT HeLa, IP 3T3L1, and IP SU-DHL1) and their union is shown in the following table. Entries in the diagonal capture the total number of proteins in the dataset.

second set<br><br>reference set	PRISMA SET1+SET2	PRISMA Core interactions	MudPIT HeLa	IP 3TL3L1	IP SU-DHL1	Union HeLa, 3T3L1, SU-DHL
**PRISMA SET1+SET2**	2363	1302	57	483	1014	1179
**PRISMA core interactions**	1302	1302	35	390	716	829
**MudPIT HeLa**	69	46	118	23	43	118
**IP 3T3L1**	447	372	23	630	349	630
**IP SU-DHL1**	904	685	41	339	1369	1369
**Union (HeLa, 3T3L1, SU-DHL1)**	1078	807	118	630	1369	1721

FDR calculation

To estimate the false discovery rate (FDR) of protein-protein interaction (PPI) data sets, we employed the method proposed by D'haeseleer and Church (2004) which relies on comparing the intersections of two measured datasets with a reference set to approximate the number of false-positive PPIs in the measured datasets (see **figure below**). We thereby assumed that the reference set and the intersection between the two measured datasets is error-free (i.e. they contain no false-positives).

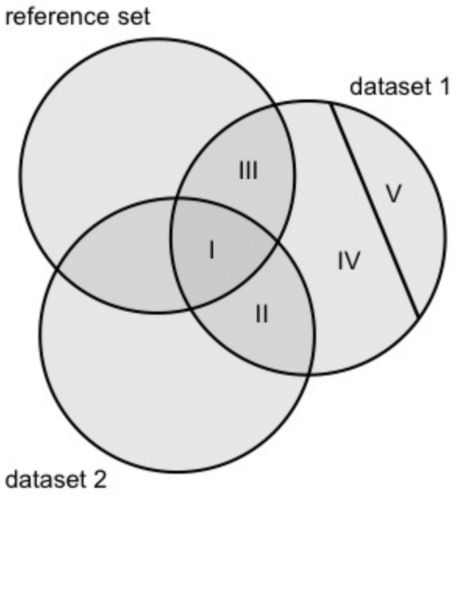

I	overlap dataset 1, dataset 2 and reference set
I U II	overlap dataset 1 and dataset 2
I U III	overlap dataset 1 and reference set
IV	true positives of dataset 1 neither in dataset 2 nor in the reference set
V	false-positives of dataset 1

$$IV / III = II / I \leftrightarrow IV = II \cdot III / I$$

$$V = |\text{data set 1}| - I - II - III - IV$$

$$FDR = V / |\text{data set 1}|$$

Scheme S1: Scheme for the calculation of the false discovery rate (FDR) of dataset 1 according to D'haeseleer & Church (2004). It is assumed that the intersections of the reference set with the datasets (I, III) as well as the intersection between the two datasets (II) are nearly error-free. In addition, the method makes use of the assumption that the reference set overlaps similarly with dataset 1 and dataset 2 (i.e. the relation IV / III = II / I holds. |dataset 1| denotes the number of proteins in dataset 1.

The assumption that the reference set is error-free can be relaxed since we only need to be sure that it contains no bias in how it intersects with the first and second measured datasets and their intersection [D'haeseleer and Church, 2004]. This can be expected if both measured datasets are obtained using the same measurement method, which is the case for PRISMA SET1 and SET2.

Using the calculation procedure described in **the figure above** for PRISMA SET1 and SET2 with the union of the IP SU-DHL1, IP 3TL3L1 and MudPIT HeLa datasets as reference set, we estimated FDRs of 11.2% and 13.9% for SET1 and SET2, respectively. If using as the two measured datasets the restrictions of SET1 and SET2 to proteins also occurring in the PRISMA core interaction set, the FDRs were reduced to 2.5% (SET1∩PRISMA core interactions) and 3.3% (SET2∩PRISMA core interactions). Overlap counts of the PRISMA sets and the reference set (union of IP SU-DHL1, IP 3TL3L1, MudPIT HeLa) which are needed for FDR calculation (i.e. values for the sizes of areas marked by I, II, III, IV, V in the figure above) are given in the following table.

	**Set size**	**I**	**II**	**III**	**IV**	**V**	**FDR**
**SET1**	1896	862	522	186	113	213	11.2%
**SET2**	1851	862	522	131	79	257	13.9%
**SET1∩PRISMA core interactions**	1203	738	359	51	25	30	2.5%
**SET1∩PRISMA core interactions**	1196	738	359	40	19	40	3.3%

Ranking of CORUM complexes

We ranked the 1432 complexes obtained from the CORUM database as described in the main text by applying a combination of two criteria: (i) the percentage of proteins of the complex occurring in PRISMA, and (ii) deviation from randomness of the coverage obtained for PRISMA and SU-DHL1. Specifically, for (i), we calculated the percentage for each complex by dividing the number of proteins occurring in the complex from the combined PRISMA replicates 1 and 2 (SET1 + SET2) with matched identifiers using the Perseus tool by the total number of proteins in the complex. For (ii), for each complex, we compared the PRISMA (SU-DHL1) dataset with random protein sets in terms of how many proteins of the complex are covered. Focusing on CORUM data, we considered only the subset of PRISMA and SU-DHL proteins which occurred in the 2678 proteins from the 1432 CORUM complexes, comprising 816 (490) proteins in the PRISMA (SU-DHL) dataset, thus also giving the size of the corresponding random datasets. We first performed the calculations separately for PRISMA and SU-DHL for each of the 1432 complexes. For calculation of a complex of *Cn* >1 proteins, of which *Cp* proteins with 0 ≤*Cp* ≤*Cn* occur in PRISMA, we estimated the probability of observing at least *Cp* of *Cn* specific proteins within a random set of size *Pn* = 816 drawn from the total set of *Tn* = 2678 proteins occurring in any of the CORUM complexes. This is a 'drawing without replacement' scenario (corresponding to the Fisher's exact test), for which success is represented by drawing one of a specific subset (of size *Cn*) of proteins, and thus the probability that at least X successes are obtained can be calculated as follows:

$$P(X \geq Cp) = \sum_{C=Cp}^{Cn} \frac{\binom{Cn}{C}\binom{Tn-Cn}{Pn-C}}{\binom{Tn}{Pn}}$$

where *Cn* is the maximal number of successes (full coverage of a complex of size *Cn*), *C* is the number of successes (a minimum of *C* = *Cp* proteins of the complex covered because this is how PRISMA or SU-DHL performed, and a maximum of *C*=*Cn*), *Tn* = 2678 (the number of possible results for drawing, which is the number of different proteins in the 1432 CORUM

complexes), and *Pn* (the number of draws or size of the random datasets) = 816 (for PRISMA full) and 490 (for SU-DHL1).

For calculation of the probabilities for different complexes, the values of *Cn* and *Cp*, but not of *Tn* and *Pn*, can change. The probabilities correspond to a hypergeometric distribution, and we employed the appropriate *R* base function (dhyper) to compute the values.

The obtained probabilities for each complex, one for PRISMA and one for SU-DHL1, represent how probable it is to obtain the observed (or a more extreme) coverage by chance. We treated the events for PRISMA and SU-DHL1 as independent and multiplied the two probabilities for each complex to obtain the probability that both coverages (or a more extreme) occurred by chance.

We ranked the 1432 complexes based on criterion (i) and (ii) separately. For (i), a high rank corresponded to a high percentage; for (ii), a high rank corresponded to a high significance (i.e. to a low probability; function rank in *R*, average values for ties). We computed the final ranking from the sum of the two previous rankings (applying minimal values for ties). The complex list is shown together with the probabilities and rankings in Supplemental Table 5.

Bibliography

Cox, J., and Mann, M. (2008). MaxQuant enables high peptide identification rates, individualized p.p.b.-range mass accuracies and proteome-wide protein quantification. Nat Biotechnol *26*, 1367-1372.

Cox, J., Neuhauser, N., Michalski, A., Scheltema, R.A., Olsen, J.V., and Mann, M. (2011). Andromeda: a peptide search engine integrated into the MaxQuant environment. J Proteome Res *10*, 1794-1805.

Csardi G, N.T.T.I., Complex Systems 1695. 2006. http://igraph.org (2006). he igraph software package for complex network research,. http://igraphorg.

D'haeseleer P, Church GM, 2004, Estimating and improving protein interaction error rates. Proceedings of the 2004 IEEE Computational Systems Bioinformatics Conference (CSB 2004), 0-7695-2194-0/04

Kanashova, T., Popp, O., Orasche, J., Karg, E., Harndorf, H., Stengel, B., Sklorz, M., Streibel, T., Zimmermann, R., and Dittmar, G. (2015). Differential proteomic analysis of mouse macrophages exposed to adsorbate-loaded heavy fuel oil derived combustion particles using an automated sample-preparation workflow. Anal Bioanal Chem *407*, 5965-5976.

Kramer, A., and Schneider-Mergener, J. (1998). Synthesis and screening of peptide libraries on continuous cellulose membrane supports. Methods Mol Biol *87*, 25-39.

Mudunuri, U., Che, A., Yi, M., and Stephens, R.M. (2009). bioDBnet: the biological database network. Bioinformatics *25*, 555-556.

Rappsilber, J., Mann, M., and Ishihama, Y. (2007). Protocol for micro-purification, enrichment, pre-fractionation and storage of peptides for proteomics using StageTips. Nature protocols *2*, 1896-1906.

Szklarczyk, D., Franceschini, A., Wyder, S., Forslund, K., Heller, D., Huerta-Cepas, J., Simonovic, M., Roth, A., Santos, A., Tsafou, K.P., *et al.* (2015). STRING v10: protein-protein interaction networks, integrated over the tree of life. Nucleic Acids Res *43*, D447-452.

Villanueva, C.J., Vergnes, L., Wang, J., Drew, B.G., Hong, C., Tu, Y., Hu, Y., Peng, X., Xu, F., Saez, E., *et al.* (2013). Adipose subtype-selective recruitment of TLE3 or Prdm16 by PPARgamma specifies lipid storage versus thermogenic gene programs. Cell Metab *17*, 423-435.

Wickham, H. (2009). ggplot2. Elegant Graphics for Data Analysis.
